# Sequence Variation of *Candida albicans* Sap2 Enhances Fungal Pathogenicity via Complement Evasion and Macrophage M2‐Like Phenotype Induction

**DOI:** 10.1002/advs.202206713

**Published:** 2023-05-21

**Authors:** Lan Lin, Moran Wang, Jingsi Zeng, Yehong Mao, Renjie Qin, Jun Deng, Xiaohu Ouyang, Xiaoshuang Hou, Chunyan Sun, Yadan Wang, Yaohua Cai, Mingyue Li, Chunxia Tian, Xi Zhou, Min Zhang, Heng Fan, Heng Mei, Alexey Sarapultsev, Huafang Wang, Gensheng Zhang, Peter F. Zipfel, Yu Hu, Desheng Hu, Shanshan Luo

**Affiliations:** ^1^ Department of Integrated Traditional Chinese and Western Medicine Union Hospital Tongji Medical College Huazhong University of Science and Technology 1277 Jiefang Avenue Wuhan 430022 China; ^2^ Institute of Hematology Union Hospital Tongji Medical College Huazhong University of Science and Technology 1277 Jiefang Avenue Wuhan 430022 China; ^3^ Department of Dermatology Union Hospital Tongji Medical College Huazhong University of Science and Technology Wuhan 430022 China; ^4^ Department of Neurosurgery Tongji Hospital Tongji Medical College Huazhong University of Science and Technology Wuhan 430000 China; ^5^ Russian‐Chinese Education and Research Center of System Pathology South Ural State University 76, Lenin Prospekt Chelyabinsk 454080 Russia; ^6^ Department of Critical Care Medicine Second Affiliated Hospital Zhejiang University School of Medicine Hangzhou Zhejiang 310009 China; ^7^ Department of Infection Biology Leibniz Institute for Natural Product Research and Infection Biology Hans Knöll Institute 07745 Jena Germany; ^8^ Faculty of Biological Sciences Friedrich Schiller University 07743 Jena Germany

**Keywords:** *Candida albicans*, complement evasion, fungal pathogenicity, immunosuppressed cellular environment, sequence variation

## Abstract

*Candida albicans* (*C. albicans*) is an opportunistic pathogen increasingly causing candidiasis worldwide. This study aims to investigate the pattern of systemic immune responses triggered by *C. albicans* with disease associated variation of Sap2, identifying the novel evasion strategies utilized by clinical isolates. Specifically, a variation in clinical isolates is identified at nucleotide position 817 (G to T). This homozygous variation causes the 273rd amino acid exchange from valine to leucine, close to the proteolytic activation center of Sap2. The mutant (Sap2‐273L) generated from SC5314 (Sap2‐273V) background carrying the V273L variation within Sap2 displays higher pathogenicity. In comparison to mice infected with Sap2‐273V strain, mice infected with Sap2‐273L exhibit less complement activation indicated by less serum C3a generation and weaker C3b deposition in the kidney. This inhibitory effect is mainly achieved by Sap2_273L_‐mediated stronger degradation of C3 and C3b. Furthermore, mice infected with Sap2‐273L strain exhibit more macrophage phenotype switching from M0 to M2‐like and more TGF‐*β* release which further influences T cell responses, generating an immunosuppressed cellular microenvironment characterized by more Tregs and exhausted T cell formation. In summary, the disease‐associated sequence variation of Sap2 enhances pathogenicity by complement evasion and M2‐like phenotype switching, promoting a more efficient immunosuppressed microenvironment.

## Graphical abstract

V273L variation of Sap2 is identified in clinical isolates. Sap2_273L_ variant enhances fungal pathogenicity. Sap2_273L_ variant more efficiently modulates complement activation by potent degradation of C3/C3b. Sap2_273L_ variant further triggers potent immunosuppressive T cell responses. Sap2_273L_ variant triggered immunosuppression of T cell responses are caused by induction of macrophage M2‐like phenotype switch.

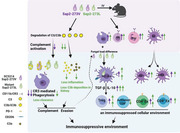

## 1 Introduction

*Candida albicans* (*C. albicans*) is a frequently isolated fungal pathogen in clinical settings and is currently causing co‐infections in COVID‐19 patients.^[^ ^1^ ^]^ Infected patients exhibit diverse genetic variations and resistance mechanisms.^[^ ^2^ ^]^ Although antifungal agents are used, systemic infections still have a high incidence rate of approximately 35%, with more than 70% mortality due to *C. albicans* infection.^[^ ^3^ ^]^ The number of drug‐resistant strains has been rapidly increasing, while the development of vaccines and specific antifungal drugs is lagging behind.^[^ ^4^ ^]^ Therefore, a comprehensive analysis of candidiasis is necessary to understand the evasion strategies and provide therapeutic guidance against fungal infection.

Fungal infections often result in complex immune responses. Researches have reported the fungal immune responses mediated by complement, or Th17 cells.^[^ ^5^ ^]^ Typically, following a fungal infection, the host complement system‐as the central innate immune surveillance system, is activated within seconds, producing powerful immune effector molecules that initiate fast cellular clearance. Molecules at the C3 level, such as surface deposited C3b/iC3b and released C3a, are essential for controlling fungal infections, as demonstrated for *C. albicans* in this study. These molecules can mediate fast fungal clearance by neutrophils and macrophages and further adaptive immune responses.^[^ ^6^ ^]^ Serum C3a can initiate inflammatory responses and also display direct antifungal effects.^[^ ^7^ ^]^ Furthermore, Th17 plays a critical role in controlling superficial candidiasis.^[^ ^8^ ^]^ To survive and establish an infection, *C. albicans* has developed several strategies to avoid complement attack. These include secreting proteases to degrade complement system factors,^[^ ^9^ ^]^ utilizing surface proteins such as Gpd2 and Gpm1 to acquire human plasma proteins and complement regulators to the fungal surface to block complement activation,^[^ ^9^, ^10^ ^]^ and secreting endogenous inhibitors to complex C3 and block further C3 cleavage by C3 convertase.^[^ ^9b,d^ ^]^ Additionally, *C. albicans* can inhibit host immune responses by modulating macrophages in cases of tumor radiotherapy.^[^ ^11^ ^]^

Sap2 plays diverse roles during *C. albicans* infection.^[^ ^12^ ^]^ For example, Gropp et al. reported that Sap1,2,3, mainly Sap2, degrade C3b, C4, and C5 of the complement system, thereby inhibiting complement activation.^[^ ^9c^ ^]^ Additionally, other studies have found that Sap2 can degrade extracellular matrix (ECM) components to breach physical barriers, aiding in the spread of infection,^[^ ^13^ ^]^ and mediate the adherence to and invasion of endothelial cells by pathogenic *C. albicans*.^[^ ^14^ ^]^ Furthermore, Sap2 is often detected in infected patients with up‐regulated secretion levels during the infection process.^[^ ^15^ ^]^ These findings underscore the importance of the Sap2 protein in clinical pathogenicity.

Microbial virulence factors frequently undergo sequence variations to evade host immune surveillance.^[^ ^16^ ^]^ Sap2, a key immune‐modulated virulence factor, displays multiple biological functions to facilitate fungal invasion. In this study, we aimed to analyze the sequence variation of *C. albicans* Sap2 in different clinical isolates derived from both superficial and systemically infected patients, further to investigate how such disease‐associated sequence variations of Sap2 modulate fungal pathogenicity by genetically engineering Sap2‐273V strain, utilizing in vivo mouse models, hopefully to identify potential variation sites closely correlated with the fungal pathogenicity, and ultimately provide therapeutic guidance against fungal infections.

## 2 Results

### 2.1 Sequence Variation of *C. albicans SAP2* in Clinical Isolates

**Figure 1:**
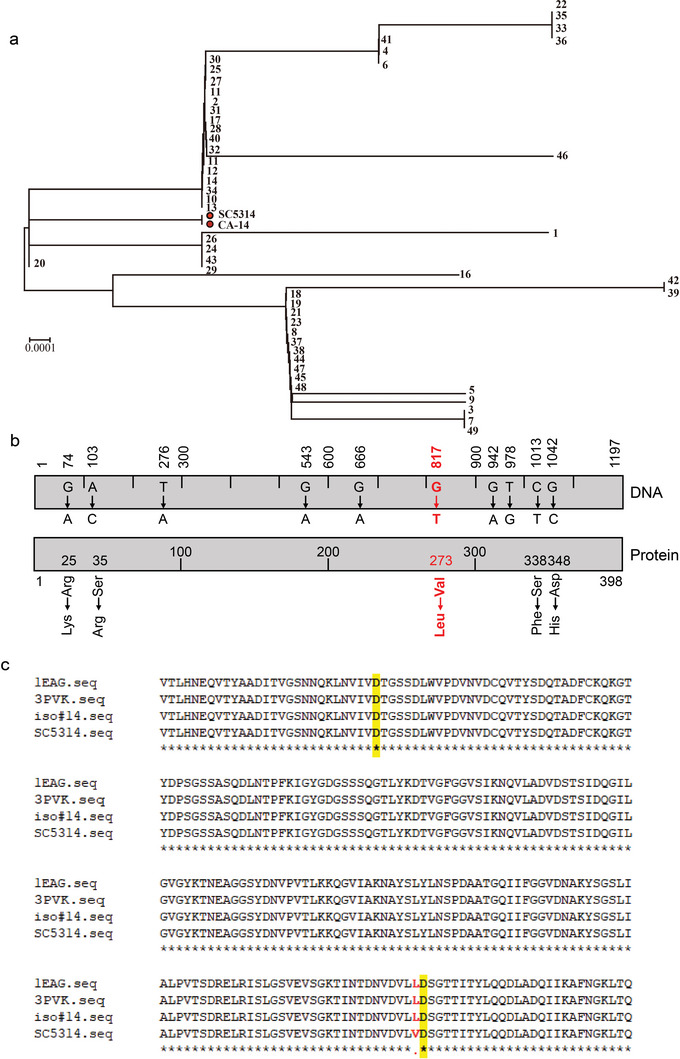
*SAP2* sequence variations of *C. albicans* clinical isolates. a) The patchy distribution of *SAP2* variants among 49 clinical isolates. b) Summary of total nucleotide exchanges within the 1197 nucleotide long *SAP2* gene and amino acid exchanges in the Sap2 protein identified among 49 clinical isolates. The mutation at nucleotide position 817 (G to T, highlighted in red) existed in all isolates tested, which caused the amino acid exchange at position 273 (valine to leucine). c) Sap2 consists of two active sites, located at 88th aa or 274th aa, respectively, and sequencing results show that the clinically identified variant is located at 273rd aa.

Microbial virulence factors frequently undergo sequence variations to evade host immune surveillance.^[^ ^16^ ^]^ In this study, we analyzed the sequences of *SAP2*, a multifunctional virulence factor, in 49 clinical isolates from patients (**Table** **1**) with superficial or systemic *C. albicans* infection and compared them with those in SC5314 and CA14 strains. The phylogenetic distribution of *SAP2* variants revealed the evolutionary relatedness of the 49 clinical isolates. The tested *SAP2* genes shared 91.88–99.67% nucleotide sequence identities, and no significant phylogenetic differences were detected among these 49 isolates. However, all 49 clinical isolates differed from the two reference strains, SC5314 and CA14 (**Figure** **1**a). The sequence variations and related amino acid exchanges compared to the reference strains are shown in Figure 1b. Overall, ten nucleotide exchanges were identified in the 1197 base pair *SAP2* gene, of which five were synonymous and five were non‐synonymous compared to SC5314. Among the five non‐synonymous exchanges, G74A, A103C, C1013T, and G1042T had a relatively low exchange rate of 2%. However, G817T variation was identified in all 49 clinical isolates, resulting in the Val273Leu variation in the protein sequence (Figure 1b).

**Table 1:** Sequence variations of clinical isolated *C. albicans SAP2*

Isolates	Sampling sites Superficial | Systemic		Number of mutations	817 G to T
1	Leucorrhea		5	Yes
2	Leucorrhea		4	Yes
3	Coronal Sulcus		4	Yes
4	Thigh		2	Yes
5	Phlegm		4	Yes
6	Leucorrhea		2	Yes
7	Thigh		4	Yes
8	Leucorrhea		2	Yes
9	Vulva Dandruff		3	Yes
10	Leucorrhea		1	Yes
11	Foreskin		1	Yes
12	Toe		1	Yes
13	Leucorrhea		1	Yes
14	Neck		1	Yes
15	Leucorrhea		2	Yes
16	Leucorrhea		4	Yes
17	Leucorrhea		1	Yes
18	Leucorrhea		3	Yes
19	Neck		3	Yes
20	Toes		1	Yes
21	Occipital Dander		3	Yes
22	Oral Cavity		2	Yes
23	Leucorrhea		3	Yes
24	Leucorrhea		3	Yes
25	Vulva		1	Yes
26	Leucorrhea		3	Yes
27	Leucorrhea		1	Yes
28	Leucorrhea		1	Yes
29	Penis		3	Yes
30	Glans		1	Yes
31	Guttural Gap		1	Yes
32	Leucorrhea		1	Yes
33	Nail Dust		2	Yes
34	Toe		3	Yes
35	Leucorrhea		4	Yes
36	Leucorrhea		1	Yes
37	Glans		2	Yes
38		Blood	3	Yes
39		Phlegm	4	Yes
40		Phlegm	1	Yes
41		Blood	2	Yes
42		Blood	1	Yes
43		Kidney	3	Yes
44		Blood	3	Yes
45		Phlegm	3	Yes
46		Phlegm	3	Yes
47		Phlegm	3	Yes
48		Blood	3	Yes
49		Phlegm	4	Yes

To understand the biological relevance of the Val273Leu variation in clinical *C. albicans*, we evaluated the model structure of Sap2_273V_ and Sap2_273L_ based on published structure data from the Protein Data Bank.^[^ ^17^ ^]^ Sap2 contains two catalytic active sites located at the 88th or 274th aa. The identified clinical variation, the 273rd aa, is close to one of the functional centers (Figure 1c and Figure S1a,b, Supporting Information). Further comparison of the model structures of Sap2_273V_ and Sap2_273L_ showed that the peptide chains of Sap2_273L_ secreted by the clinical isolates are closer than Sap2_273V_ secreted by SC5314 (Figure S1b, Supporting Information), suggesting the possibility of altered activity of the protease.

To confirm this, we generated and purified two forms of recombinant proteins (Sap2_273V_ and Sap2_273L_). The purity and quality of both recombinant proteins were quantified by Coomassie staining and western blotting using a specific anti‐Sap2 antibody. Our data revealed that both Sap2 proteins were identified as 42 kDa bands with over 95% purity (Figure S1c, Supporting Information). Next, we analyzed and compared the protease activity of purified Sap2_273L_ and Sap2_273V_ using an Invitrogen EnzChek Protease Assay Kit. The results showed that both Sap2_273V_ and Sap2_273L_ strongly degraded casein at pH 5, compared to the buffer control. However, Sap2_273L_ exhibited significantly stronger proteolytic activity than Sap2_273V_. These results were also observed at pH 7 (Figure S1d, Supporting Information).

Moreover, we analyzed the effects of these residues on Sap2‐mediated degradation of known substrates C3 and C3b. Purified Sap2_273V_ and Sap2_273L_ were incubated with either C3 or C3b at pH 5 and pH 7, and the degradation products were detected by western blotting using specific anti‐C3 antibodies. The results demonstrated that Sap2_273L_ exhibited a much stronger ability to degrade C3 than Sap2_273V_ at pH 5. Sap2_273L_ almost completely degraded the *α*‐chain of C3 and partially degraded the *β*‐chain, while Sap2_273V_ showed a weaker effect when an equal amount of Sap2 was applied. A similar pattern was observed for C3b degradation mediated by Sap2_273L_ and Sap2_273V_. The incubation at pH 7 produced similar results but with weaker intensity (Figure S1e, Supporting Information). Therefore, the Val273Leu variation likely modulates the Sap2 catalytic activity. However, additional in vitro and in vivo assays are required to demonstrate whether and how such a sequence variation enhances fungal pathogenicity by modulating Sap2 biological function.

### 2.2 The Site‐Directed Mutant Displayed Elevated Fungal Pathogenicity

**Figure 2:**
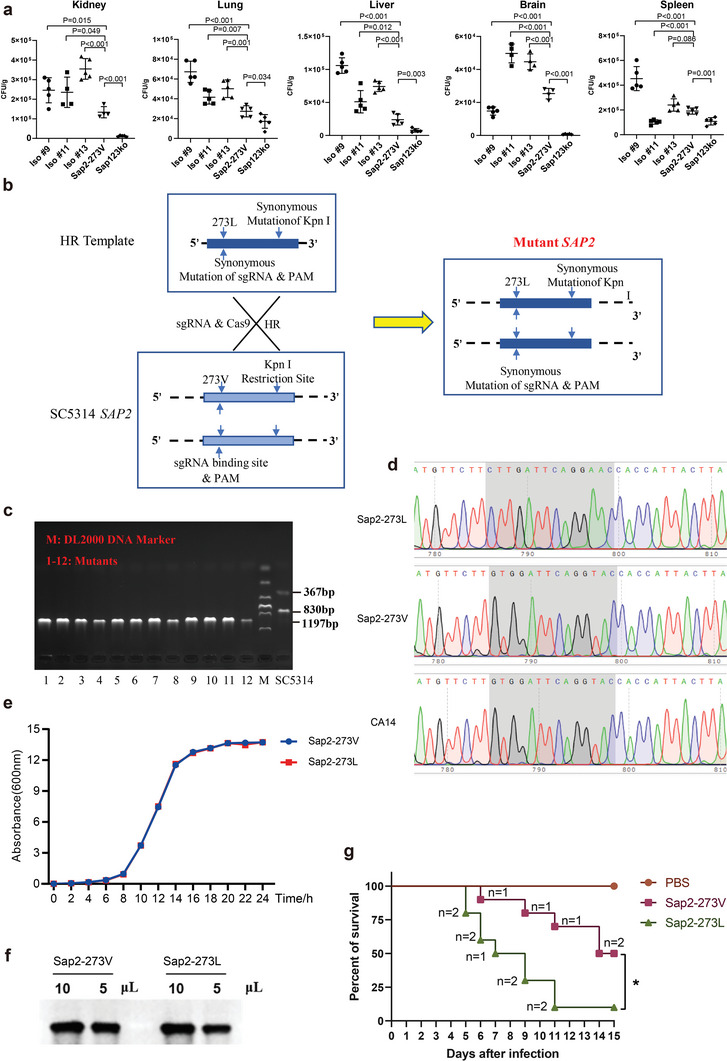
The genetically engineered Sap2‐273L displayed elevated fungal pathogenicity. a) Three different clinical isolates as well as Sap2‐273V and Sap123 ko strains were selected and intravenously injected into wild type C57Bl6 mice (*n* = 5 per group, 5 × 10^5^ CFU/mouse). Mice were sacrificed at 16 h post infection, kidney, lung, liver, brain, and spleen were therefore taken and homogenized. Samples were pre‐diluted and plated on YPD agar plates, then cultivated at 30 °C for 48 h. The colonies were counted and compared among different groups. b,c) Generation of mutant strain carried V273L variation within Sap2. CRISPR‐cas9 was used to mutate the 817th site within the *SAP2* gene from G to T by recombining the template into the genome of the Sap2‐273V strain. d) Sanger sequencing was performed to identify the *SAP2* gene of the three strains. e) The growth rate of the Sap2‐273L and Sap2‐273V strains. The equal growth rate of the two strains was observed. f) Sap2 secretion levels by the Sap2‐273L and Sap2‐273V strains were detected by the specific Sap2 antibody. g) The survival rate of mice infected with the Sap2‐273L and Sap2‐273V strains (2 × 10^5^ CFU/mouse) for 15 days, ten mice per group, “*n*” indicates the number of mice dead in a given day. Data are shown as means ± SD, and from one of three independent experiments. The survival rate of mice was analyzed by a Kaplan–Meier log rank test, and the *p* values were calculated by the Student's *t*‐test.

To establish a basis for further research, we first examined whether the identified sequence variation affected fungal pathogenicity in general. We assessed the fungal burden caused by clinical isolates and compared it to that caused by the reference strain SC5314 (further referred as Sap2‐273V), as it is a common indicator of microbial virulence. Upon intravenous infection of mice with clinical *C. albicans* isolates (Iso#9, 11, 13) harboring single or multiple mutations of 5 × 10^5^ CFU (100 µL/mouse) for 16 h, more severe fungal loads were observed in most organs (i.e., kidneys, lung, and liver) compared to Sap2‐273V, indicating that clinical isolates displayed higher virulence upon infection. However, unequal distribution of Iso #9 and Iso #11 was detected in the brain and spleen, respectively (**Figure** **2**a), likely due to the polymorphism of other virulence factors present in clinical isolates, such as Pra1.^[^ ^16b^ ^]^ Therefore, a site‐directed mutant strain originating from the Sap2‐273V should be employed for further analysis.

CRISPR‐cas9 is a widely used method to create site‐specific mutations in fungal strains.^[^ ^18^ ^]^ In order to investigate the relevance of the Val273Leu variation on fungal pathogenicity in vivo, we utilized CRISPR‐cas9 to introduce a homozygous mutation (G817T) into Sap2‐273V, generating a site‐mutated strain that carried Sap2_273L_ (further referred as Sap2‐273L) (Figure 2b). Enzymatic digestion and sequencing confirmed the successful introduction of the mutation (Figure 2c,d). The growth rate and Sap2 secretion level of Sap2‐273V and Sap2‐273L strains were found to be similar (Figure 2e,f). Upon infection of mice with Sap2‐273V, Sap2‐273L strain (2 × 10^5^ CFU, 100 µL/mice), or PBS, Sap2‐273L infected mice exhibited higher mortality rates, with 25% of mice dying on day 5 and over 90% dying by day 13 post‐infection, while all Sap2‐273V infected mice survived at day 5 and less than 20% died by day 13 (Figure 2g).

Organ damage was examined in three groups of mice infected with Sap2‐273V, Sap2‐273L strain, or PBS (1 × 10^5^ CFU, 100 µL/mouse) for 12 days. The kidney exhibited pathological changes in both fungal‐infected groups compared to the PBS‐treated group, with more severe damage detected in Sap2‐273L infected mice (Figure S2a, Supporting Information). Moreover, a higher fungal load was observed in the kidneys of Sap2‐273L infected mice during long‐term infection (Figure S2b, Supporting Information). These results demonstrate that the genetically engineered site‐directed mutated Sap2‐273L exhibited significantly higher fungal pathogenicity than Sap2‐273V.

### 2.3 The Site‐Directed Mutant Modulated Complement Activation In Vivo

**Figure 3:**
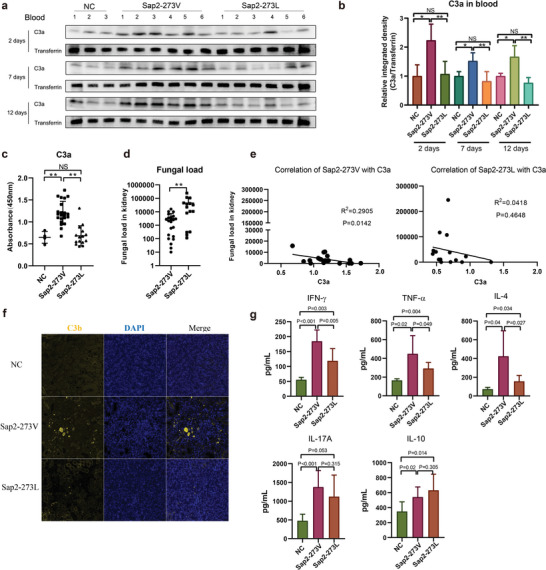
Genetically engineered Sap2‐273L strain potentially modulated complement activation and early inflammatory cytokine pattern. a,b) C3a level in the blood of fungal infected mice. Mice were intravenously infected with Sap2‐273L (*n* = 6), Sap2‐273V (*n* = 6) strains, or PBS (*n* = 3) (1 × 10^5^ CFU/mouse). At day 2, day 7, and day 12 post infection, C3a in the blood was detected by western blotting and compared by integrated density. c–e) Correlation between blood C3a level and fungal burden in mice. Mice were intravenously infected with Sap2‐273L (*n* = 15), Sap2‐273V strains (*n* = 20), or PBS (*n* = 5) (1 × 10^5^ CFU/mouse) and sacrificed on day 2 post infection. Blood levels of C3a were detected by ELISA (c), the fungal burden in the kidney was counted and compared between groups (d), and then the correlation between blood C3a level and fungal burden was analyzed (e). f) C3b deposition (yellow) was detected by immunofluorescent staining of the kidney slides. g) The levels of different cytokines released in the serum of fungal infected mice. Mice were intravenously injected with the same amount of Sap2‐273L, Sap2‐273V strains, or PBS (*n* = 8 per group) (1 × 10^5^ CFU/mouse). The cytokines in serum at day 2 post infection were detected by cytometric bead array. Data are shown as means ± SD, and from one of three independent experiments. *p* values were analyzed by Student's *t*‐test, *p < 0.05, **p < 0.01.

As Sap2‐273L strain exhibited significantly increased fungal pathogenicity, further in vivo experiments were conducted to examine the differences in host systemic immune responses elicited by Sap2‐273L or Sap2‐273V strain. In early responses, the complement system serves as the central immune surveillance mechanism for managing pathogenic infections. Notably, complement activation products at the C3 level play crucial roles in controlling fungal infections, including C3b/iC3b‐mediated phagocytosis and C3a‐mediated antifungal activity.^[^ ^19^ ^]^ Consequently, our primary focus was to analyze C3a release and C3b deposition to quantify complement activation in mice upon infection. Our findings revealed that mice infected with Sap2‐273V strain demonstrated significantly elevated C3a release both in serum and kidney on day 2, 7, and 12 post‐infection (1 × 10^5^ CFU, 100 µL/mouse) (**Figure** **3**a,b and Figure S3a,b, Supporting Information, lines 4–9), respectively. Conversely, reduced C3a levels were observed in Sap2‐273L‐infected mice (Figure 3a,b and Figure S3a,b, Supporting Information, lines 10–15). Given the higher fungal burdens (Figure S2, Supporting Information) and decreased complement activation observed in Sap2‐273L infections, we sought to investigate the correlation between C3a levels and fungal loads in the kidney following infection with Sap2‐273L, Sap2‐273V, or PBS (1 × 10^5^ CFU, 100 µL/mouse) on day 2. Interestingly, we discovered a negative correlation between serum C3a levels and fungal burden in the kidney, particularly in the Sap2‐273V‐infected group, with a minor correlation detected in the Sap2‐273L‐infected group (Figure 3c–e). This may be attributable to the excessive degradation of C3 already initiated during the early infection phase, therefore causing C3a levels in the Sap2‐273L‐infected group to remain consistently low throughout the infection process. Altogether, these results indicated that Sap2‐273L strain induced significantly stronger complement inhibitory effects than Sap2‐273V strain, a phenomenon attributable to the Sap2_273L_ variant.

Additionally, C3b/iC3b deposition was investigated using immunofluorescent staining. The results demonstrated that Sap2‐273L infected mice displayed reduced C3b/iC3b deposition in the kidney compared to Sap2‐273V infected ones (Figure 3f). Owing to the potent suppression of complement activation, we hypothesized that the site‐directed Sap2‐273L infection might also result in diminished inflammation during the early infection phase by evading host complement attack. Consequently, the pro‐ and anti‐inflammatory cytokines released in the serum on day 2 post‐infection with Sap2‐273V, Sap2‐273L, or PBS (1 × 10^5^ CFU, 100 µL/mouse) were examined. Our findings indicated that lower levels of proinflammatory cytokines, such as IFN‐*γ*, TNF‐*α*, and IL‐4, were detected in mice infected with Sap2‐273L strain compared to those infected with Sap2‐273V strain. Conversely, the serum levels of IL‐17A and IL‐10 were elevated in both Sap2‐273L and Sap2‐273V‐infected mice, while with no statistical differences observed (Figure 3g). These results suggested that Sap2‐273L strain more effectively evaded early immune responses, including complement activation and inflammation, thereby facilitating increased fungal accumulation in vivo.

### 2.4 Sap2‐273L Caused Enhanced Inhibitory Effect on Complement Activation Was Mainly Achieved by Sap2_273L_‐Mediated Potent Degradation of C3/C3b

Sap2 is a type of aspartate protease that has been reported to cleave various components of the complement system, including C3b, C4, C5, and factor H, at pH 5, indicating its potential non‐specificity.^[^ ^9c^ ^]^ Our in vivo findings showed that infection with the site‐directed mutated Sap2‐273L strain in mice led to reduced release of C3a and deposition of C3b/iC3b. This suggests that the inhibitory effect on complement C3 levels may have been achieved through stronger degradation of C3/C3b by Sap2_273L_. To confirm this, we used purified recombinant Sap2_273V_ and Sap2_2 73L_ and incubated them with C3/C3b at different concentrations (0.25, 0.5, 1, and 2 µg) to visualize the degradation products through western blotting. Our data showed that both Sap2_273L_ and Sap2_273V_ effectively degraded C3 in a dose‐dependent manner, with the *α*‐chain gradually decreasing in intensity and degradation products appearing in the lower part of the gel. However, Sap2_273L_ showed a stronger ability to degrade C3 compared to Sap2_273V_, as indicated by the different intensity of the C3 *α*‐chain when an equal amount of Sap2 was applied. At a dosage of 2 µg, Sap2_273L_ almost completely degraded the *α*‐chain of C3 and even partially degraded the *β*‐chain, while Sap2_273V_ had a weaker effect. Similar results were observed for C3b degradation mediated by both Sap2_273L_ and Sap2_273V_. However, the intensity of the degradation products was not gradually increased as expected. It is possible that the progressive degradation of small products was also triggered by both Sap2 proteins. These data suggest that Sap2_273L_ may enhance the ability of Sap2 to degrade complement C3 and C3b.

**Figure 4:**
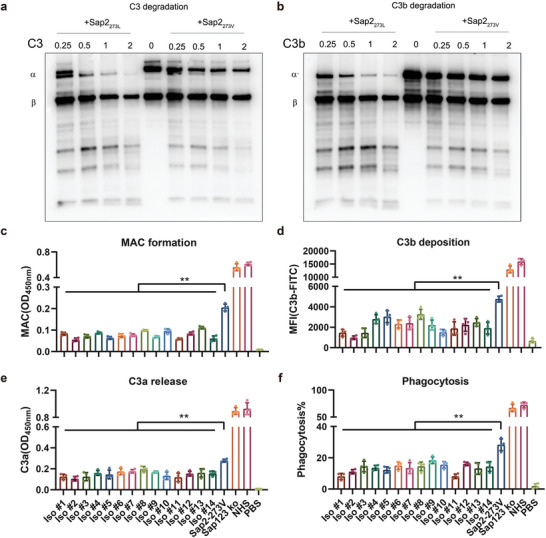
Site mutated Sap2_273L_, by potentially degrading C3 and C3b, displayed stronger complement inhibitory effects. a,b) Sap2 mediated concentration‐dependent degradation of C3/C3b. Different doses (0.25, 0.5, 1, 2 µg) of purified Sap2_273V_ and Sap2_273L_ proteins were incubated with purified human C3 or C3b (0.5 µg), afterward, the mixtures were separated by SDS‐PAGE under reducing conditions, and the degradation products of C3 (a) and C3b (b) were detected by western blotting using polyclonal goat anti human C3 antibody. c) Effect of V273L variation of Sap2 on MAC formation, PBS indicated a negative background control without NHS in the sample. d) Effect of V273L variation of Sap2 on C3b/iC3b deposition, PBS indicated a negative background control without NHS in the sample. e) Effect of V273L variation of Sap2 on C3a release, PBS indicated a negative background control without NHS in the sample. f) Effect of V273L variation of Sap2 on C3b/iC3b mediated phagocytosis of *C. albicans* by THP‐1. The phagocytosis of Sap2‐273V strain by THP‐1 cells was quantified by flow cytometry as double positive cells (FITC^+^, DiD^+^), PBS indicated a negative background control without *C. albicans*. Data are shown as means ± SD, and from one of three independent experiments. *p* values were analyzed by Student's *t*‐test, **p < 0.01.

To further confirm that the Sap2_273L_ variant exhibits stronger complement inhibitory effects than Sap2_273V_, potentially due to greater degradation of complement C3 and C3b, in vitro analyses were conducted using Sap2‐containing supernatants from various clinical isolates, as well as Sap2‐273V strain. Initially, multiple clinical isolates harboring two or more variations (Iso #1 through #9) or a single V273L variation (Iso #10 through #14), in addition to Sap2‐273V and Sap123 knockout (ko) strains, were cultured. The Sap2 secretion levels in the supernatants were then analyzed and pre‐quantified using a specific anti‐Sap2 antibody (Figure S3c,d, Supporting Information). Subsequently, normal human serum (NHS) was incubated with varying volumes of supernatant containing an equal amount of pre‐calculated Sap2 based on Sap2 concentration and further activated. Membrane attack complex (MAC) formation, C3a release, and C3b/iC3b surface deposition, which represent complement activation, were subsequently quantified. Compared to the non‐treated control, Sap2_273V_ secreted by Sap2‐273V strain significantly inhibited MAC formation by approximately 67%, while Sap123 ko did not affect MAC formation. Notably, when compared to Sap2_273V_ secreted by Sap2‐273V strain (set as 100%), Sap2_273L_ secreted by clinical isolates (Iso #1 through Iso #14) further inhibited MAC formation by approximately 59.5%, 73.0%, 65.5%, 57.8%, 69.0%, 63.9%, 62.7%, 52.0%, 66.6%, 53.4%, 71.2%, 59.0%, 45.9%, and 70.4%, respectively (**Figure** **4**c). Similarly, Sap2_273L_ secreted by various clinical strains or Sap2‐273L strain demonstrated more potent inhibition of C3b/iC3b surface deposition and C3a release compared to Sap2_273V_ secreted by Sap2‐273V strain, while Sap123 ko exhibited no inhibitory effect (Figure 4d,e and Figure S3e, Supporting Information). These results further substantiate that Sap2_273L_ variant enhances Sap2‐mediated inhibitory effects on complement activation.

As phagocytes can recognize C3b/iC3b opsonized on microbial pathogen surfaces through the CR3 receptor to mediate pathogen phagocytosis,^[^ ^20^ ^]^ and the Sap2_273L_ variant enhances Sap2‐mediated inhibition of C3b/iC3b surface deposition, we hypothesized that the Sap2_273L_ variant also modulates C3b/iC3b‐mediated phagocytosis. Flow cytometry analysis revealed that Sap2_273V_ secreted by Sap2‐273V strain obstructed C3b/iC3b‐mediated adhesion and phagocytosis of *C. albicans* by THP‐1 cells by approximately 60.9% compared to the NHS control, while Sap123 ko exhibited no inhibitory effects. Notably, in comparison to Sap2_273V_ secreted by Sap2‐273V strain (set as 100%), Sap2_273L_ secreted by 14 clinical isolates (Iso #1 through Iso #14) further impeded phagocytosis by roughly 71.6%, 60.9%, 49.2%, 51.9%, 56.8%, 47.2%, 52.6%, 48.7%, 35.0%, 45.3%, 71.3%, 43.4%, 53.6%, and 49.3%, respectively (Figure 4f and Figure S3f,g, Supporting Information). However, no statistical difference was observed among the tested isolates, indicating that Sap2 with single or multiple variations exhibited similar effects. These findings suggest that Sap2 indeed blocked phagocytosis of *C. albicans* by human macrophages, and the Sap2_273L_ variant further enhanced this inhibitory effect. In summary, these results demonstrated that the Sap2_273L_ variant, by potently degrading complement C3 and C3b, more effectively inhibited host complement activation and complement‐mediated phagocytosis.

### 2.5 The Site‐Directed Mutated Sap2‐273L Strain Further Triggered Potent Immunosuppressive T Cell Responses

**Figure 5:**
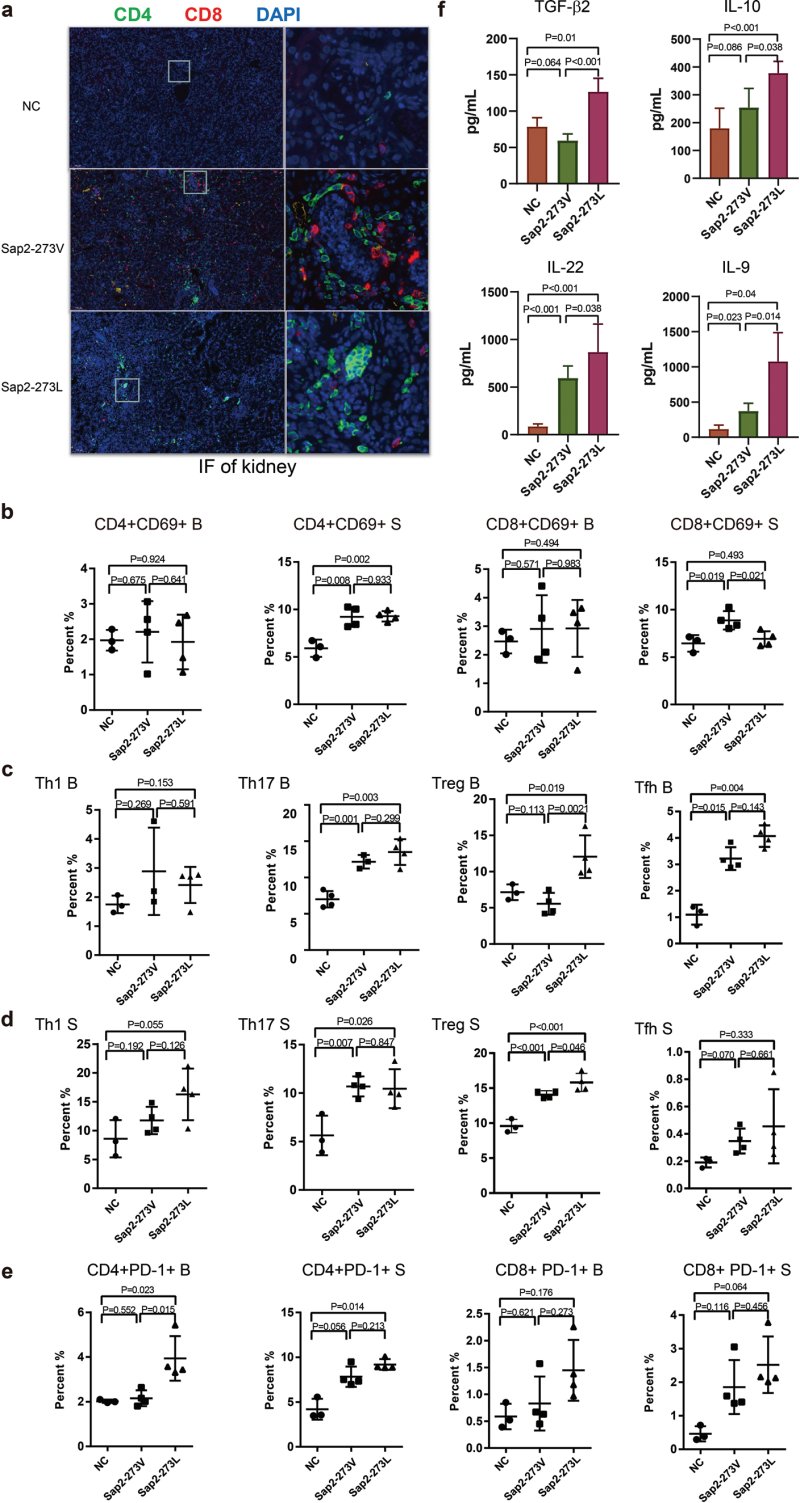
Genetically engineered Sap2‐273L strain triggered a stronger immunosuppressed microenvironment. Mice were intravenously injected with an equal amount of Sap2‐273L (*n* = 4), Sap2‐273V strains (*n* = 4), or PBS (*n* = 3) (1 × 10^5^ CFU/mouse). At day 12 post infection, a) CD4^+^/CD8^+^ T cells (green/red) infiltration and C3b deposition (yellow) were detected by immunofluorescent staining of the kidney. b) The early activation of CD4^+^/CD8^+^T cells in B (blood) and S (spleen) was analyzed by flow cytometry. CD4^+^CD69^+^ population indicated the early activation of CD4^+^ T cells, and CD8^+^CD69^+^ population was early activation of CD8^+^ T cells. c,d) The differentiation of CD4+ T help cells in the B (blood) (c) and S (spleen) (d) analyzed by flow cytometry. Th1 cells, Th17, and Treg cells were classified by the positive expression of T‐bet, ROR*γ*T, and FoxP3. Tfh cells were identified by the high expression of CXCR5 and PD‐1. e) The exhaustion of CD4^+^/CD8^+^ T cells in B (blood) and S (spleen) was detected by expression of immune checkpoint molecule PD‐1. f) The levels of different cytokines released in serum at day 12 post infection. Mice were intravenously infected with the same amount of Sap2‐273L, Sap2‐273V strains, or PBS (*n* = 8 per group) (1 × 10^5^ CFU/mouse). The cytokines in serum were detected by cytometric bead array. Data are shown as means ± SD, and from one of three independent experiments. The *p* values were calculated by the Student's *t*‐test.

Numerous studies have demonstrated that distinct immune responses occur following *C. albicans* infection, illustrating that innate immune responses govern the early response, primarily mediated by complement activation, succeeded by adaptive immune responses such as Th17 cell responses.^[^ ^5^, ^6^ ^]^ Given the significant difference in pathogenicity between Sap2‐273L and Sap2‐273V strains observed in our mouse infection models, in addition to the differences detected in complement activation, we hypothesized that adaptive immune responses might also differ between site‐directed mutated Sap2‐273L and Sap2‐273V infected mice (1 × 10^5^ CFU, 100 µL/mouse). To test this, we first examined CD4^+^ and CD8^+^ T cell infiltration in the kidneys using immunofluorescent staining. The results revealed that Sap2‐273L strain induced lower CD8^+^ T cell infiltration compared to Sap2‐273V strain (**Figure** **5**a). Next, we analyzed changes in effector and memory T cell populations both in the spleen and blood using flow cytometry on day 12 post‐infection. No marked differences were observed between the two infection groups in either blood or spleen (Figure S4a,b, Supporting Information). However, when evaluating CD69 expression on CD8^+^ or CD4^+^ T cell surfaces to monitor early T cell activation, we found that early CD8^+^ T cell activation was significantly lower in Sap2‐273L infected mice than in Sap2‐273V infected mice. In contrast, early CD4^+^ T cell activation increased in the spleens of both fungal infection groups, with no discernible difference between them (Figure 5b and Figure S4c, Supporting Information). These findings indicate that site‐directed mutated Sap2‐273L strain elicited fewer CD8^+^ T cell responses in mice compared to Sap2‐273V strain.

It is well‐established that CD8^+^ T cell activation relies on CD4^+^ helper T cells in the spleen. Consequently, we further investigated CD4^+^ T cell differentiation in both infection groups. The percentages of Th17, Treg, and Tfh cells increased for both fungal infected groups, in line with previous reports.^[^ ^5^, ^21^ ^]^ Intriguingly, Treg cells were more abundant in the blood and spleen of Sap2‐273L infected group compared to the Sap2‐273V infected group. In contrast, Th1 cells did not exhibit a significant increase in the control group in our infection model (Figure 5c,d and Figure S4d,e, Supporting Information). Additionally, we analyzed and compared PD‐1 expression on T cells between the two infection groups to investigate T cell exhaustion. The findings suggested that Sap2‐273L strain might induce greater exhausted T cell formation, as higher PD‐1 expression was detected in Sap2‐273L infected group compared to the Sap2‐273V infected one, particularly for CD4^+^ T cells in the blood (Figure 5e).

In order to further confirm the pattern of cell differentiation, the release of cytokines was examined in mice infected with the fungus. The induction of TGF‐*β*, IL‐10, IL‐9, and IL‐22 indicated a specific antifungal immune response. Additionally, the levels of IL‐10, TGF‐*β*, IL‐22, and IL‐9 were found higher in mice infected with the Sap2‐273L strain than in those infected with Sap2‐273V strain (Figure 5f), which was consistent with the direction of T cell differentiation. To better demonstrate the relative virulence of the two fungal strains, a detailed kinetic study was performed to dynamically monitor the fungal load in various organs, C3a levels, and immune profiling on days 2, 7, and 12 post‐infection. Similar patterns were observed. In summary, Sap2‐273L infected mice displayed higher fungal load overall, less C3a release all the time, more exhausted CD4^+^ T cells, Treg cells and M2 macrophage induced, as well as less pro‐inflammatory cytokines released, but more IL‐10 and IL‐9 production at day 12 post‐infection (Figures S4f and S5, Supporting Information). Therefore, *C. albicans* utilized Sap2 to induce Treg differentiation, exhausted T cell formation, and the secretion of TGF‐*β* and TGF‐*β* associated cytokines, such as IL‐10, IL‐9, and IL‐17, to suppress host T cell responses. Sap2‐273L strain induced a more potent immunosuppressive environment than Sap2‐273V strain.

### 2.6 *Candida albicans* Triggered Immunosuppression of T Cell Responses Were Caused by Induction of Macrophage M2‐Like Phenotype Switch

**Figure 6:**
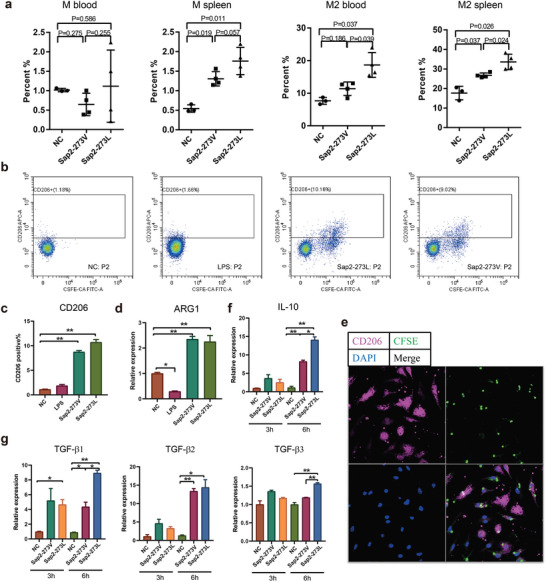
*C. albicans* triggered immunosuppressed T cell responses by inducing macrophage M2‐like phenotype switch. a) The types of macrophages in blood and spleen were examined by flow cytometry using CD11c, CD11b, and F4/80^+^ as detection markers, *n* = 4 per group. b,c) The expression of CD206 in RAW cells stimulated by Sap2‐273L, Sap2‐273V strains, or LPS was detected by flow cytometry. d) The cytokine ARG‐1 level was detected by qPCR. e) Co‐localization of BMDM cells (CD206^+^, pink) and *C. albicans* (CFSE^+^, green) was detected by immunofluorescent staining. f,g) IL‐10 and TGF‐*β* transcription level upon co‐culture of RAW cells with Sap2‐273L or Sap2‐273V for 3 and 6 h. Data are shown as means ± SD, and from one of three independent experiment. *p* values were analyzed by Student's *t*‐test. *p < 0.05, **p < 0.01.

Antigen‐presenting cells (APCs) are the primary cell types that differentiate CD4^+^ T cells, with macrophages, non‐lymphoid conventional dendritic cells (cDCs), and B cells presenting antigens to T cells for their activation and differentiation.^[^ ^22^ ^]^ In our infection models, DCs (cDC1 and cDC2), B cells, neutrophils, and NK cells in both fungal infected groups were slightly elevated but did not show any significant difference between the two infected groups (Figure S6a–c, Supporting Information). However, the percentage of macrophages was significantly higher in the spleen of both infected groups and displayed an even higher level in Sap2‐273L infected mice than in Sap2‐273V infected ones (**Figure** **6**a). Further analysis revealed that the number of CD206^+^ macrophages (M2 type) was also substantially higher in Sap2‐273L infected group than in the Sap2‐273V infected ones (Figure 6a,b and Figure S6d–f, Supporting Information). This finding is closely correlated with the patterns of Tregs and exhausted T cell formation (Figure 5c–e), indicating that the more M2 cells were triggered, the more Tregs and exhausted T cells were generated.

A previous report has suggested that long‐term *C. albicans* infection can trigger M2 macrophage transformation,^[^ ^7^ ^]^ and since there was no difference in other APCs observed, macrophages are the most likely candidates mediating Treg formation in our fungal infection mouse models. However, it was unclear how *C. albicans*, by triggering M2 macrophage formation, further induced Treg formation. Interestingly, we found that CD11b expression was elevated on the surface of M2 cells upon macrophage phenotype switch in both infection groups (Figure S6e, Supporting Information). Previous reports have shown that soluble *β*‐glucan secreted by *C. albicans* binds to CD11b on the surface of monocytes and induces the release of TGF‐*β* transporting vesicles.^[^ ^23^ ^]^ We hypothesized that such an interaction pattern further stimulates significantly higher TGF‐*β* release by M2 macrophages. To test this, we utilized an in vitro infection model. We analyzed CD206^+^ and Arg‐1 expression upon co‐culture of RAW and bone marrow‐derived macrophages (BMDM) cells with Sap2‐273L and Sap2‐273V strains, and fluorescence analysis showed that high CD206^+^ expressing cells were surrounded by the fungi, and CD206 was co‐localized with *C. albicans* (Figure 6e). All three factors (CD206, Arg‐1, and IL‐10) were highly up‐regulated compared to the non‐treated control (Figure 6c,d,f). Next, we analyzed cytokine expression patterns by fungal‐treated macrophages using qPCR and ELISA. We found that TGF‐*β* was significantly increased in both *C. albicans* infected cells. Meanwhile, Sap2‐273L strain triggered even more TGF‐*β* release (mainly TGF‐*β*2) at 6 h post infection (Figure 6g and Figure S7b, Supporting Information). Additionally, M1‐type cytokines, such as IL‐4, IL‐12, IL‐6, IL‐23, TNF‐*α*, IL‐1*β*, and IFN‐*β*, were slightly elevated upon stimulation by fungi, but no statistical difference was detected between the two fungal infection groups (Figure S7a–c, Supporting Information). These data indicate that fungal infection triggers an M2‐like phenotype switch (but not a typical M2 type), releasing TGF‐*β* and thereby assisting further immunosuppressive responses. The Sap2‐273L strain displays a stronger ability to induce an M2‐like phenotype switch, thereby triggering more potent immunosuppressive responses.

**Figure 7:**
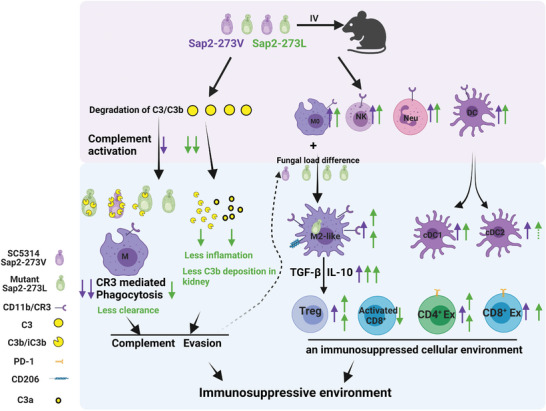
The model of how disease associated V273L variation within *Candida albicans* Sap2 enhances fungal pathogenicity. Our study illustrated the V273L variation, by enhancing Sap2‐mediated C3 and C3b cleavage, efficiently blocked C3b/iC3b surface deposition and CR3‐mediated phagocytosis. This chain of events led to a more potent deficiency of the fast clearance of Sap2‐273L strain. Therefore, persistent chronic infection of Sap2‐273L strain with a higher fungal burden in the body accelerated the phenotype switch of macrophages from M0 to M2‐like type, which in turn up‐regulates CD11b expression level on the surface of M2‐like macrophages. A higher CD11b level, by binding to more *β*‐glucan present on fungal surfaces, triggered M2‐like macrophages to potentially release TGF‐*β*. Such cytokine patterns further interfered with T cell responses and developed an immunosuppressed microenvironment characterized by more Tregs and exhausted T cell formation.

Overall, our study demonstrated that the Sap2_273L_ variant efficiently blocked C3b/iC3b surface deposition and CR3‐mediated phagocytosis by enhancing Sap2‐mediated C3 and C3b cleavage. This resulted in a more potent deficiency of complement‐mediated fast clearance of Sap2‐273L strain. Consequently, persistent chronic infection caused by Sap2‐273L strain with a higher fungal burden in the body accelerated the phenotype switching of macrophages from M0 to M2‐like type, with an up‐regulation of CD11b expression level on the cell surface, further potentially releasing TGF‐*β*. Such cytokine patterns further interfered with T cell responses and developed an immunosuppressed cellular microenvironment characterized with more Tregs and exhausted T cell formation (**Figure** **7**).

## 3 Discussion and Conclusions

*C. albicans* is the major cause of fungal bloodstream infection which can lead to clinical symptoms such as sepsis and septic shock, with high morbidity and mortality worldwide. Upon pathogen invasion, innate immune responses govern the early response, mainly through complement activation, followed by adaptive immune responses, such as Th17 cell responses.^[^ ^5^, ^6^ ^]^ Fungal pathogens employ different strategies to block and silence immune responses in order to cause infection. In this process, pathogenic virulence factors that mediate immune evasion play vital roles in initiating severe infection. Therefore, characterizing these evasion proteins in clinical isolates or highly pathogenic strains enables the identification of novel evasion strategies as well as possible targets against microbial pathogens. Investigating the systemic immune response is also of great importance in revealing the novel strategies utilized for fungal infection.

Sap2 is a major virulence factor of *C. albicans* that modulates complement activation by degrading C3b, C4, and C5, as well as ECM to escape physical barriers for tissue invasion.^[^ ^9^, ^24^ ^]^ Sap2 has been detected in multiple organs and tissues infected by *C. albicans*,^[^ ^15a^ ^]^ and its expression level is up‐regulated upon infection.^[^ ^15b^ ^]^ The Sap2 protein itself has strong immunogenicity and can induce mucosal and systemic antibody responses. A specific antibody against Sap2 has been detected in people who were ever infected by *C. albicans*.^[^ ^25^ ^]^ Therefore, changes in the immunogenicity of *C. albicans* Sap2 may help it evade host immune attack. In this work, we analyzed sequence variations of *SAP2* at the DNA and protein level in 49 clinical *C. albicans* isolates for the first time and further characterized how these sequence variations contribute to fungal pathogenicity by modulating host immune responses.

Microbial immune evasion proteins can be divided into two significant classes. The first group encodes immune evasion proteins with conserved sequence repertoires, and the individual proteins show minor sequence variation. The second group of evasive proteins show higher sequence diversity. For example, *C. albicans* Pra1 exhibits a high degree of sequence variability at the DNA and protein levels, with a mutation rate of 1.8%.^[^ ^16b^ ^]^ Among other microbial virulence factors that mediate complement evasion, CRASPs proteins of *Borrelia* species have been reported as moderately conserved proteins.^[^ ^26^ ^]^ In contrast, the M protein of *S. pyogenes* and the PspC protein family of *S. pneumonia* were highly polymorphic and divergent.^[^ ^16a^ ^]^ In this work, we analyzed 1197 bp of the *C. albicans SAP2* gene and identified ten variations, representing five non‐synonymous and five synonymous mutations, with an allele exchange rate ranging from 0.02 to 1.0. Notably, among the ten detected variations, the G817T exchange results in the replacement of Val with Leu at position 273, occurring in all detected isolates with an allelic frequency of 1.00.

When reviewing the database in the UniProt, we noticed a missense variant of the 273rd amino acid site reported for Sap2. Upon further searching for the article that reported this variant,^[^ ^27^ ^]^ we found that the sequence information provided for *SAP2* was for CA14, but not SC5314. They reported a heterozygous mutation from G to T at position 817 of the *SAP2* gene in one chromosome of CA14. We were confused why it was inconsistent with our sequencing result of the *SAP2* gene of the CA14. To answer the confusion, we further investigated the genomic information for SC5314 (https://www.ncbi.nlm.nih.gov/genome/gdv/browser/gene/?id=3647354) and the lineages of *C. albicans* (http://www.candidagenome.org/Strains.shtml#SC5314). The genome annotation and recent sequencing results indicate that *SAP2* gene of SC5314 is indeed non‐mutated and homozygous. Since CA14 was evolved from SC5314 under the pressure of 5FO,^[^ ^28^ ^]^ we assume that CA14 may appear mutation or non‐mutation divergence within *SAP2* in order to accommodate survival under selection pressure. Microbial pathogens undergo sequence variation to represent antigenic diversity, ultimately allowing for better invasion and increased survival. Therefore, the G817T mutation, occurring either under selection pressure, in the clinic, or during evolution, provides insight into the importance of this special site (817) for fungal pathogenicity.

The model structural analysis indicated that the Val273Leu variant was located near one of the two activation sites of Sap2, and the peptide chain spindle for Sap2_273L_ was narrower than that of Sap2_273V_ secreted by SC5314. These findings suggested that the Val273Leu variation may influence protein functions and fungal pathogenicity. Protease activity assays were performed using known substrates for Sap2, and the results demonstrated that Sap2_273L_ exhibited stronger proteolytic activity than Sap2_273V_. To investigate the in vivo relevance of the Val273Leu variation on fungal pathogenicity, CRISPR‐cas9 was used to mutate the 817th site within *SAP2* from G to T based on Sap2‐273V strain, to generate a mutant strain that carried the Val273Leu variation. In the in vivo infection model, it was observed that although the Sap2‐273L and Sap2‐273V strains had almost equal growth rates and similar Sap2 secretion levels, the fungal burden was significantly higher in mice infected by Sap2‐273L than by Sap2‐273V strain. Furthermore, mice infected with Sap2‐273L strain displayed a higher mortality rate after a longer period of infection, indicating that the Val273Leu variant enhances fungal pathogenicity and exacerbates *C. albicans* virulence.

Upon the initial fungal infection, innate immune responses take the lead followed by a slower‐acting adaptive immune response. Therefore, we first examined complement activation and early inflammatory responses in our fungal infection model to observe any differences in the innate immune response elicited by the genetically engineered Sap2‐273L strain and the reference strain Sap2‐273V. Interestingly, we found that mice infected with the Sap2‐273L strain showed significantly reduced (or even absent) complement activation represented by reduced C3a release and C3b deposition in the kidney, as well as decreased production of pro‐inflammatory cytokines. Molecular analysis conducted in vitro showed that such inhibitory effects on complement activation and complement‐mediated phagocytosis were primarily caused by the potential degradation of complement C3 and C3b by the Sap2_273L_ variant. As complement‐mediated phagocytosis is essential for the fast clearance of microbial pathogens during infection, Sap2_273L_‐mediated stronger inhibitory effects on complement activation and complement‐mediated phagocytosis resulted in more significant defects in fast clearance of *C. albicans*, leading to a higher fungal burden and persistent chronic infection in the host.

*C. albicans*’ Sap2 is an aspartate protease that can cleave a wide range of protein targets, including ECM proteins, kininogens, and proteins of the complement system (such as C3b, C4, C5, and factor H) in vitro at pH 5.^[^ ^29^ ^]^ The complexity of Sap2's functions makes studying its effects in vivo challenging. Our focus in this study is to analyze how disease‐associated sequence variation of Sap2 modulates host systemic immune responses, including complement activation and adaptive immune responses. Specifically, we investigated the effects of the Val273Leu variant on C3 and C3b degradation, and on in vivo C3a release and C3b/iC3b deposition, as complement activation products at the C3 level are crucial for controlling fungal infection. However, we cannot exclude the possibility that the Val273Leu variant also affects ECM proteins, kininogens, C4, C5, and Factor H cleavage.

In our in vivo and in vitro models, we found that persistent chronic fungal infection leads to up‐regulation of CD206 and CD11b on the macrophage surface. The greater the fungal load, the more CD206 is expressed, resulting in an M2‐type phenotype switch. Higher CD11b levels can bind more *β*‐glucan on *C. albicans*' surface, which may trigger macrophages to release significantly higher levels of TGF‐*β*. Such cytokine patterns released by M2‐type macrophages further interfere with T cell responses and create an immunosuppressive microenvironment characterized by more Tregs and exhausted T cell formation, as previously reported.

Our in vitro infection models revealed that in addition to the potent M2‐type cytokines TGF‐*β* and IL‐10, M1‐type cytokines such as TNF‐*α*, IL‐1*β*, and IL‐6 were also slightly induced upon fungal infection. However, no statistical differences were observed between the Sap2‐273L and Sap2‐273V infected groups (Figure S7a–c, Supporting Information). These data indicate that both types of macrophages were induced upon *C. albicans* infection, and the CD206^+^ macrophages are most likely M2‐like macrophages, rather than typical M2‐type. The formation of M2‐like macrophages creates an immunosuppressive environment, allowing fungal proliferation and systemic fungal infection. This phenotype switching is similar to that observed in previous reports on tumor‐associated macrophages characterized by high expression of IL‐10 and TGF‐*β* and low expression of inflammatory cytokines due to delayed activation of NF‐*κ*B, creating an immunosuppressive environment that facilitates tumor progression.^[^ ^30^ ^]^

This study is the first to investigate the disease‐associated sequence variation of *C. albicans* Sap2 and its clinical relevance in fungal pathogenicity. We found that the Sap2_273L_ variant enhances fungal virulence by blocking complement activation and inducing a macrophage M2‐like phenotype switch, ultimately leading to a potent immunosuppressive T cell response. These findings shed light on a novel evasion strategy employed by *C. albicans* to escape host immune surveillance during systemic infections and may guide the development of effective treatments for candidiasis. Furthermore, due to the formation of Tregs and exhausted T cells during systemic fungal infection, immune activation reagents such as anti‐PD‐1 or PD‐L1 may be promising candidates for efficient fungal eradication.

## 4 Experimental Section

### Ethics Statements

This study was approved by the Ethical Board of Tongji Medical College, Huazhong University of Science and Technology, China (Ethics code: S071). Written consent was obtained from patients for participation in the study. In total, 49 strains were isolated from dermatological or systemic fungal infected patients using FungiQuick swabs. The swabs were incubated in Sabouraud glucose bouillon for 24 h at room temperature (RT). Then, the bouillon yeast was plated onto Sabouraud glucose agar plates and further incubated for 24 h at 30 °C. According to the manufacturer's instructions, the cultures obtained were used for species identification using the API ID 32C test system (bioMerieux). The identified *C. albicans* were used for further analysis.

6‐week‐old male C57BL/6 mice were housed under specific pathogen‐free conditions at the Animal Care Center of Tongji Medical College, Huazhong University of Science and Technology (Wuhan, China) for the animal experiments. This study followed the Guidelines for the Care and Use of Laboratory Animals of the National Institutes of Health, and all protocols were approved by the Institutional Animal Care and Use Committee of Tongji Medical College (Permit Number: 2595).

### Growth Conditions of *Candida albicans* Strains

The *C. albicans* strains SC5314 (referred as Sap2‐273V strain in this work)^[^ ^31^ ^]^ and various clinical isolates were cultured in the YPD medium, including 2% w/v glucose, 2% w/v peptone, and 1% w/v yeast extract, for 16 h at 30 °C.^[^ ^10^ ^]^ The cultured yeast cells were collected by centrifugation, and the density was measure by a spectrophotometer.

### Antibodies and Serum

The polyclonal rabbit anti‐Sap2 antibody was kindly provided by Prof. Bernhard Hube (HKI Jena, Germany). Polyclonal goat anti‐human C3 was purchased from CompTech, and polyclonal rabbit anti‐MAC was obtained from Abcam. HRP‐labeled rabbit anti‐goat, goat anti‐rabbit, and Alexa Fluor‐488‐labeled donkey anti‐goat antibodies were purchased from Abcam. NHS was collected from five healthy donors, pooled together, and stored at −80 °C for further use.

### Sequence Analysis

The *SAP2* gene isolated from clinical isolates and the reference strain Sap2‐273V were amplified by PCR, sequenced, and aligned. Briefly, yeast pellets of different *C. albicans* strains were boiled at 99 °C for 10 min in ddH_2_O, shaken at 300 rpm, and finally centrifuged at 13 000 rpm for 10 min to obtain the supernatant containing chromosomal DNA. The obtained chromosomal DNA was used as a template. Thereafter, the *SAP2* gene was amplified using specific primers (Forward: 5′‐AATCAATCAAATAACAACAACCCAC‐3′, Reverse: 5′‐TTTTAATATTTTAACTTTATTCCACCCC‐3′. The PCR products were first separated by the agarose gel electrophoresis, then the bands were visualized under the UV detector (GENE GENIUS 1, SYNGENE). Subsequently, the proved PCR products were purified using the GFX purification kit (GE Healthcare) and sequenced with 3730× 1 Genetic Analyzer (AB Applied Biosystems).

### Enrichment of *Candida albicans* Sap2, Generation and Purification of Recombinant Sap2_273V_ and Sap2_273L_

An enriched medium was applied to induce Sap2 secretion. Briefly, Sap2‐273V strain, Sap123 ko, and the clinical isolates were first cultured in YPD medium overnight at 30 °C, then the pellets (2 × 10^7^ CFU/sample) of each strain were further incubated in 1 mL of Sap2 enriched medium, 0.25% BSA (Sigma), 1% glucose, 7.3 mm KH_2_PO_4_, 2 mm MgSO_4_, and 1% 100 minimum essential medium vitamins (pH 4.0) for 48 h at 30 °C to induce Sap2 expression.^[^ ^9^, ^32^ ^]^ For the purification of Sap2, cultured supernatant was first concentrated using Centricon Plus‐20 with a 5 kDa cutoff (Millipore Corporation, Billerica). The concentrated samples were then loaded into a protein A column pre‐coupled with anti‐BSA antibody, then additional contaminants and exchange buffer in the eluates were removed using a size exclusion column. The final elution fractions were quantified by Coomassie staining and western blotting and used for further functional analysis.

To produce the Sap2_273V_ and Sap2_273L_ recombinant proteins, the methods described in previous studies were followed.^[^ ^33^ ^]^ Briefly, the open reading frame of *SAP2* was cloned into the pET‐28a vector and expressed in *Escherichia coli* BL21 as a zymogen. After the inclusion bodies were dissolved in 8 m urea and renatured by dialysis with 10 mm Tris (pH 8.0), the supernatant was purified using a His‐tag Protein Purification Kit (Denaturant‐resistant) (BeyoGold, P2229S). The quality and purity of the purified recombinant proteins were assessed by Coomassie staining and western blotting. The endotoxin levels of the purified recombinant proteins were also measured using an endotoxin detection kit (Thermo Scientific, A39552S) to ensure that there was no endotoxin contamination

### Complement Activation Assay and Protease Activity Detection

Complement activation was carried out as previously reported.^[^ ^9^, ^10^ ^]^ Briefly, to study the effect of Val273Leu variation of Sap2 on complement activation, NHS was first pre‐treated with an equal amount of Sap2 secreted by different clinical strains, Sap2‐273V or Sap123 ko strains for 15 min at 37 °C, then added to 96‐well plates pre‐coated with lipopolysaccharide (LPS, 10 µg mL^−1^) and further incubated for 60 min at 37 °C. The MAC formation was then quantified by polyclonal rabbit anti‐MAC antibody (1:3000) together with HRP‐labeled goat anti‐rabbit as a secondary antibody (1:2000). PBS used in the assay indicated a negative background control without NHS in the sample.

Protease activity of Sap2_273V_ and Sap2_273L_ at pH 5 and pH 7 was detected using a Protease Assay Kit (Invitrogen EnzChek Protease Assay Kit). Specifically, fluorescence‐quenched BODIPY FL casein (10 µg mL^−1^) was first incubated with 1 µg recombinant Sap2_273V_ and Sap2_273L_ in PBS (pH 5 or 7) for 60 min at 25 °C, then the fluorescence was measured in a fluorescence microplate reader, using excitation and emission filters of 485 ± 12.5 and 530 ± 15 nm, respectively.

### Western Blotting

To detect Sap2 secretion levels, Sap2‐273V, Sap2‐273L, and Sap123 ko strains were first cultivated in Sap2 enriched medium. Then, equal volumes of culture supernatants were treated with Roti‐load1 for 10 min at 98 °C, separated by SDS‐PAGE, and transferred to the PVDF membrane. After blocking with 4% milk powder, Sap2 was detected using the polyclonal rabbit anti‐Sap2 antibody, followed by HRP‐labeled goat anti‐rabbit as a secondary antibody.

To analyze the effect of Val273Leu variation on Sap2 mediated degradation of human C3 and C3b, different doses (0.25, 0.5, 1, 2 µg) of purified recombinant Sap2_273V_ or Sap2_273L_ were incubated with the human C3 or C3b (0.5 µg) in PBS (total volume 50 µL, pH 5 or pH 7) for 60 min at 37 °C. C3 and C3b degradation in the mixture were then detected by western blotting using polyclonal goat anti‐human C3, followed by the secondary HRP‐labeled rabbit anti‐goat antibody.

### C3a Release and C3b/iC3b Surface Opsonization

To determine the effect of Val273Leu variation of Sap2 on C3a release and C3b/iC3b deposition, NHS (7.5 µL) was pre‐incubated with an equal amount of Sap2 secreted by different clinical isolates, Sap2‐273V, Sap2‐273L, or Sap123 ko strains at 37 °C for 15 min. PBS indicated a negative background control without NHS in the sample. The mixture was then activated by heating inactive *C. albicans* in an Mg‐EGTA buffer (100 µL) at 37 °C for 30 min. After centrifugation, the supernatant was collected to analyze C3a release using the C3a ELISA kit. Additionally, the C3b/iC3b deposition on the surface of *C. albicans* was analyzed by flow cytometry using polyclonal goat anti‐C3 and an Alexa Fluor‐488‐labeled rabbit anti‐goat as the secondary antibody.

### Phagocytosis Assay

To assay the effect of Val273Leu variation on C3b/iC3b mediated phagocytosis of *C. albicans* by macrophages, C3b/iC3b opsonized *C. albicans* were prepared as mentioned above, followed by incubation with CFSE (10 µm mL^−1^, BD, 565082) for 20 min at RT to label the *C. albicans* pellets. Meanwhile, THP‐1 cells were seeded onto a 24‐well tissue culture plate (Greiner bio‐one) in the RPMI1640 medium (1 × 10^6^/well) in 5% CO_2_ at 37 °C overnight, and then stimulated with PMA (1 µL mL^−1^) for 16 h to achieve macrophages. Subsequently, THP‐1 was labeled using Vybrant DiO (1:100, Invitrogen) for 40 min at 37 °C. Finally, CFSE‐labeled C3b/iC3b opsonized *C. albicans* were added to these THP‐1 cells and incubated for 15 min. Flow cytometry was used to measure the adhesion and phagocytosis of *C. albicans* by THP‐1 cells, and was quantified as the double‐positive fraction (DiO^+^, FITC^+^). PBS indicated a negative background control without *C. albicans* in the culture.

### Generation of Mutant Strain Carried the Val273Leu Variation within Sap2

To better evaluate the modulating effects of Val273Leu variation of Sap2 on host immune responses in vivo, the CRISPR‐Cas9 gene editing technology was applied to mutate G to T at position 817 of *SAP2* using Sap2‐273V strain (SC5314) as the background strain following previously published protocols.^[^ ^18^, ^34^ ^]^ Briefly, sgRNAs for *SAP2* were retrieved using https://benchling.com. A relatively high score of sgRNA was chosen near the 817th site. The oligos (ATTTGtgataatgtcgatgttcttgG; AAACcaagaacatcgacattatcaC) were synthesized to ligate the sgRNA into the plasmid PV1093. Subsequently, the desired mutation of G to T was introduced into the template at the 817th position, which was then transfected into Sap2‐273V strain to generate a mutant strain carrying Sap2_273L_ named Sap2‐273L strain. Furthermore, the synonymous mutation was also used to modify the PAM site to circumvent the second editing by cas9 and mutate the restriction endonuclease recognition site KpnI to facilitate screening. Thereafter, the long oligos tcagttgaagtttctggtaaaaccatcaatactgataatgtcgatgttcttcttgattcaggaaccacc and accattgaaagctttaatgatttgatcagcaagatcttgcaaataagtaatggtggttcctgaatcaagaagaac were used, and recombinantly modified template was produced by PCR. The detailed protocol was referring to the steps provided by JOVE (https://www.jove.com/cn/t/58764/crispr‐mediated‐genome‐editing‐human‐fungal‐pathogen‐candida). Differently, each colony was labeled on the transformation plate and the genome was roughly extracted for the next colony PCR. After labeling each single colony on the transformation plate, the labeled colony was picked up from the plate with a sterile tip to 30 µL buffer A (20 mL Buffer A: ddH2O 19.942 mL, 10 m NaOH 50 µL, 0.5 m EDTA 8 µL) and the samples were incubated at 95 °C for 30 min, then 30 µL buffer B (20 mL Buffer B: ddH_2_O 19.2 mL, 1 m Tris‐HCL (pH = 8.0) 800 µL) was added into the above tubes for neutralization of buffer A. After centrifugation, the supernatant could be used for the next colony PCR.

Following the positive selection, serial passages were performed until the cas9 gene couldn't be detected by PCR and Nat resistance disappeared. Then the *SAP2* in the Sap2‐273L strain was verified by sequencing using atgtttttaaagaatattttcattgctcttgctattgc and ttaggtcaaggcagaaatactggaagcag oligos. Subsequently, the growth rate and the Sap2 secretion level of the generated Sap2‐273L strain were analyzed and compared with the reference Sap2‐273V strain.

### In Vivo Mouse Experiment

To evaluate the in vivo virulence of clinical *C. albicans* isolates harboring sequence variation within *SAP2*, three selected clinical isolates, Sap2‐273V as well as Sap123 ko (5 × 10^5^ CFU, 100 µL/mouse) strains were intravenously injected into the wild type C57Bl6 mice. At 16 h post infection, the kidneys, lung, liver, brain, and spleen were collected, homogenized, and plated. The plated samples were then cultivated at 30 °C for 48 h. Finally, the colonies on the plates were counted to evaluate the fungal burden in the individual organ.

To further characterize the effect of Val273Leu variation on fungal pathogenicity, the first genetically engineered Sap2‐273L or Sap2‐273V strains (2 × 10^5^ CFU, 100 µL/mouse) were intravenously injected into C57Bl6 mice. The mice survival rate was recorded based on the number of mice death per day for a period of 15 days.

Furthermore, to analyze the modulating effects of the Sap2_273L_ variant on host immune responses in vivo, mice were intravenously infected with the Sap2‐273L or Sap2‐273V strains (1 × 10^5^ CFU, 100 µL/mouse), or PBS. At day 12 post infection, mice were sacrificed, and blood and spleen were collected to detect the phenotype changes of immune cells using flow cytometry. To better demonstrate the relative virulence status of two fungal strains, a detailed kinetic study was performed to dynamically monitor fungal load in various organs, C3a levels, and immune profiling at day 2, day 7, and day 12 post infection. Briefly, mice were intravenously injected with the Sap2‐273L, Sap2‐273V strains (1 × 10^5^ CFU, 100 µL/mouse), or PBS. At day 2, day 7, or 12 post infection, mice were sacrificed, blood and spleen were collected to detect the phenotype changes of immune cells using flow cytometry, serum was collected for cytokines detection by cytometric bead array (CBA), and kidney, liver, and brain were collected for the investigation of fungal burden. C3a release in serum and kidney was analyzed by western blotting using polyclonal rabbit anti‐C3a antibody as well as HRP‐labeled goat anti rabbit as the secondary antibody. In addition, immunohistochemical assays were used to detect C3b deposition and T cell infiltration in the kidney.^[^ ^35^ ^]^ To analyze the correlation of C3a with fungal load, the mice were infected with Sap2‐273L and Sap2‐273V strains (1 × 10^5^ CFU, 100 µL/mouse). At day 2 post infection, blood was collected to detect the C3a release by ELISA and kidneys were collected to determine the fungal burden. The correlation was analyzed by linear regression.

### Flow Cytometric Analysis of Immune Responses or Related Cytokines Released In Vivo

Flow cytometry was performed as previously described.^[^ ^36^ ^]^ For cell surface staining, cells isolated from the spleen or blood were stained with fluorescence‐labeled antibodies on ice in the dark for 20 min. The samples were then analyzed by flow cytometry after washing within 1 h. For the staining of transcript factor or CD206, cells were fixed and permeated by the transcription factor staining buffer (Thermo Fisher, 00‐5523‐00). All antibodies and reagents used for flow cytometry analysis are listed in Table S1, Supporting Information. Plasma cytokines including IL‐4, IL‐10, IL‐17A, IL‐22, IFN‐*γ*, IL‐9, and TNF‐*α* were measured using a Mu Th cytokine‐panel Kit (741043, Biolegend).

### ELISA, RT‐PCR, and IF Analysis of the Macrophage Responses upon Fungal Infection

Primary BMDM were isolated according to a procedure previously described.^[^ ^37^ ^]^ The isolated BMDM and RAW cells were infected with the same amount of the Sap2‐273L or Sap2‐273V strains. The cells were then lysate by TRizol (TAKARA) or fixed with polyoxymethylene at 3 or 6 h post infection. Subsequently, relative expression of cytokines was detected by RT‐PCR (Thermo, A25742). The primers (Actin, TGF*β*‐1, TGF*β*‐2, TGF*β*‐3, TNF‐*α*, IFN‐*γ*, IFN‐*β*, IL‐1*β*, IL‐4, IL‐6, IL‐10, IL‐12, IL‐23) used in this study are listed in Table S2, Supporting Information. In addition, TNF‐*α* and IL‐1*β* were further confirmed at the protein level using ELISA kits (eBioscience, 88‐7324‐88, eBioscience, 88‐7013‐88). CD206 expression by RAW cells was detected by flow cytometry. The colocalization of CD206 on the surface of BMDM with *C. albicans* was examined by a confocal laser scanning microscope (Biolegend‐141708).

According to the qPCR results, TGF*β*‐1 and TGF*β*‐2 appeared distinct. To further prove them in protein levels, ELISA kits were applied to further quantify the concentration of TGF*β*‐1 and TGF*β*‐2 in the serum or the culture supernatant after fungal infection according to the manual (Dakewe, 1217102, Multi Science, EK9162). The samples were activated by 1 m HCl for 10 min at RT and further neutralized by 1.2 m NaOH. The serum samples were diluted by 200 times and the cell culture supernatant was diluted by 20 times.

### Statistical Analysis

Statistical analyses were performed with the assistance of GraphPad Prism software. The two‐tailed Student's *t*‐test was performed as indicated and specifically, the survival rate of mice was analyzed by a Kaplan–Meier log rank test. The results were expressed as mean ± SD unless stated otherwise. Group sizes, reproducibility, and *p* values for each experiment are stated in figure legends.

## Conflict of Interest

The authors declare no conflict of interest.

## Author Contributions

L.L. and M.W. contributed equally to this work. S.L., D.H., Y.H., and P.F.Z. designed the study and wrote the manuscript. L.L. and M.W. performed the experiments, analyzed the data, gave conceptual advice, and wrote the manuscript. R.Q., J.D., X.O., X.H., A.S., C.S., Y.W., Y.C., M.L., C.T., and X.Z. collected and analyzed the data; J.Z., Y.M., M.Z., H.F., H.M., H.W., and G.Z. provided reagents and conceptual advice. L.L.: Conceptualization|Supporting, Data curation|Lead, Formal analysis|Lead, Funding acquisition|Supporting, Investigation|Lead, Methodology|Lead, Project administration|Supporting, Resources|Supporting, Software|Lead, Supervision|Supporting, Validation|Lead, Visualization|Lead, Writing—original draft|Equal, and Writing—review and editing|Equal. J.D.: Investigation|Supporting and Resources|Supporting. X.Z.: Data curation|Supporting and Formal analysis|Supporting. M.Z.: Investigation|Supporting. H.F.: Resources|Supporting. A.S.: Resources|Supporting. Y.H.: Conceptualization|Equal, Funding acquisition|Equal, Resources|Equal, and Writing—original draft|Equal.

## Supporting information

Supporting Information

## Data Availability

The data that support the findings of this study are available from the corresponding author upon reasonable request.
