# Supplementary material for: Sequence Variation of Candida albicans Sap2 Enhances Fungal Pathogenicity via Complement Evasion and Macrophage M2‐Like Phenotype Induction
Source: Adv Sci (Weinh). 2023 May 21;10(20):2206713. doi: 10.1002/advs.202206713 (PMC10369283; doi:10.1002/advs.202206713)
Supplement: Supplementary file 1 — Supporting Information [file ADVS-10-2206713-s001.pdf]

ADVANCED SCIENCE
Open Access

Supporting Information

for *Adv. Sci.*, DOI 10.1002/advs.202206713

Sequence Variation of *Candida albicans* Sap2 Enhances Fungal Pathogenicity via Complement Evasion and Macrophage M2-Like Phenotype Induction

Lan Lin, Moran Wang, Jingsi Zeng, Yehong Mao, Renjie Qin, Jun Deng, Xiaohu Ouyang, Xiaoshuang Hou, Chunyan Sun, Yadan Wang, Yaohua Cai, Mingyue Li, Chunxia Tian, Xi Zhou, Min Zhang, Heng Fan, Heng Mei, Alexey Sarapultsev, Huafang Wang, Gensheng Zhang, Peter F. Zipfel, Yu Hu, Desheng Hu* and Shanshan Luo**

Supplementary information

Sequence Variation of *Candida albicans* Sap2 Enhances Fungal Pathogenicity via Complement Evasion and Macrophage M2-like Phenotype Induction

Lan Lin[1a], Mo-Ran Wang[2a], Jing-Si Zeng[3], Ye-Hong Mao[3], Ren-Jie Qin[1], Jun Deng[2], Xiao-Hu Ouyang[1], Xiao-Shuang Hou[4], Chun-Yan Sun[2], Ya-Dan Wang[2], Yao-Hua Cai[2], Ming-Yue Li[1], Chun-Xia Tian[1], Xi Zhou[2], Min Zhang[2], Heng Fan[1], Heng Mei[2], Alexey Sarapultsev[5], Hua-Fang Wang[2], Gen-Sheng Zhang[6], Peter F. Zipfel[7,8], Yu Hu[2*], De-Sheng Hu[1,2*], Shan-shan Luo[2*]

L. Lin, R. J. Qin, X.H. Ouyang, M.Y. Li, C.X. Tian, H. Fan, D.S. Hu[*]

Department of Integrated Traditional Chinese and Western Medicine, Union Hospital, Tongji Medical College, Huazhong University of Science and Technology, 1277 Jiefang Avenue, Wuhan 430022, China.

M.R. Wang, J. Deng, C.Y. Sun, Y.D. Wang, Y.H. Cai, X. Zhou. M. Zhang, H. Mei, H.F. Wang, Y. Hu[*], D.S. Hu[*], S.S. Luo[*]

Institute of Hematology, Union Hospital, Tongji Medical College, Huazhong University of Science and Technology, 1277 Jiefang Avenue, Wuhan 430022, China.

J.S. Zeng, Y.H. Mao

Department of Dermatology, Union Hospital, Tongji Medical College, Huazhong University of Science and Technology, Wuhan, China

X.S. Hou

Department of Neurosurgery, Tongji Hospital, Tongji Medical College, Huazhong University of Science and Technology, Wuhan 430000, China

A. Sarapultsev

Russian-Chinese Education and Research Center of System Pathology, South Ural State University, 76, Lenin prospekt, Chelyabinsk, Russia, 454080.

G. S. Zhang

Department of Critical Care Medicine, Second Affiliated Hospital, Zhejiang University School of Medicine, Hangzhou, Zhejiang 310009, China

P.F. Zipfel

Department of Infection Biology, Leibniz Institute for Natural Product Research and Infection Biology, Hans Knöll Institute, Jena, Germany; Friedrich Schiller University, Faculty of Biological Sciences, Jena, Germany

a. These authors contributed equally to this work

Correspondence to:

Shanshan Luo, 2018xh0258@hust.edu.cn

Desheng Hu, desheng.hu@hust.edu.cn

Yu Hu, dr_huyu@126.com

Keywords: Fungal pathogenicity, *Candida albicans*, sequence variation, immunosuppressed cellular environment, complement evasion

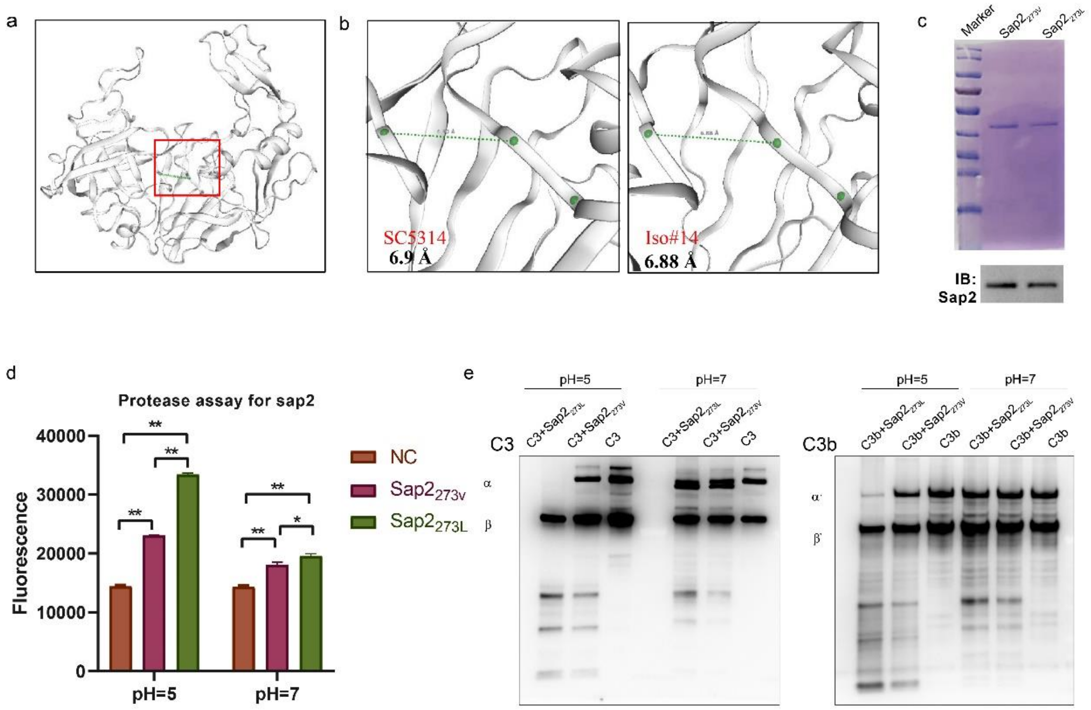

Figure S1. Structure prediction indicated that V273L variation is located close to the active center of *Candida albicans* Sap2 and affects the protease activity. (a) Prediction of the model structures of Sap2 secreted by Sap2-273V strain and the clinical isolates based on the published protein structures. Sap2 consists of two activation sites, which are located either at the 88th aa or 274th aa, while the clinically identified variation of Sap2 is at 273th aa. **(b)** Comparison of the model structures of $Sap2_{273V}$ and $Sap2_{273L}$. The 273rd aa is close to one of the functional centers. (**c**) Detection of the purity of recombinant $Sap2_{273V}$ and $Sap2_{273L}$ by coomassie staining and western blotting. (**d**) Protease activity of $Sap2_{273V}$ and $Sap2_{273L}$ at pH 5 and pH 7 was detected according to the Invitrogen EnzChek Protease Assay Kit. The evolving fluorescence was measured at 485 nm excitation/525 nm emission after 60-120 mins of reaction at 25°C in a microplate reader. (**e**) The effect of V273L variation on Sap2 mediated degradation of C3 and C3b was detected at pH 5 or pH 7. Purified recombinant $Sap2_{273V}$ and $Sap2_{273L}$ proteins (2ug) were incubated with purified human C3 or C3b (0.5 ug), afterwards, the degradation products of C3 (left) and C3b (right) were detected

by western blotting. Data are shown as means ± SD and from one of three independent experiments.

*$P < 0.05$, **$P < 0.01$, Student’s *t*-test.

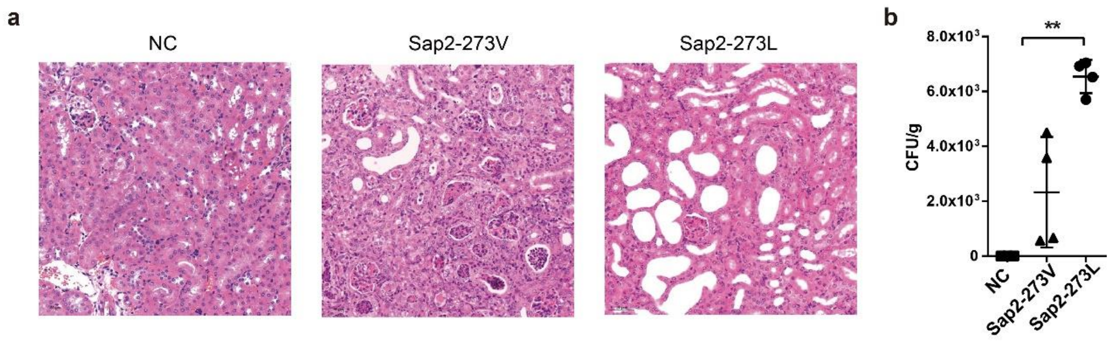

Figure S2. Tissue damage and fungal burden of the kidney upon *C. albicans* infection. Mice were intravenously infected with the same amount of Sap2-273V (n=4), Sap2-273L strains (n=4), or PBS (n=3), (1 x 10^5 CFU/mouse) and sacrificed on day 12 post infection. (**a**) H&E staining of the kidney. (**b**) The fungal loads in the kidney were calculated by plating. Data are shown as means ± SD and from one of three independent experiments. ***P* < 0.01, Student's *t*-test.

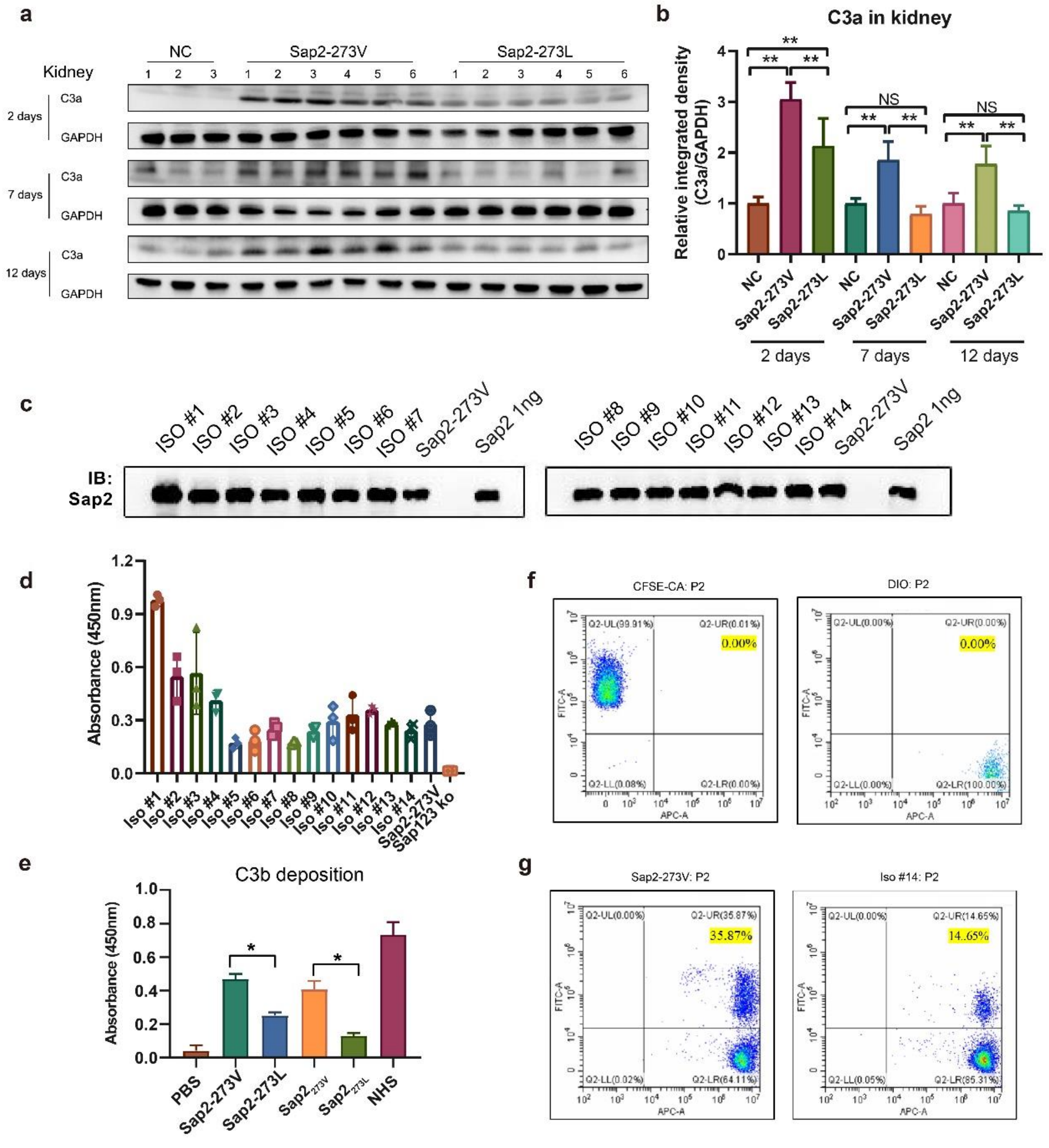
a
Kidney
NC
Sap2-273V
Sap2-273L
2 days
7 days
12 days
C3a
GAPDH
b
C3a in kidney
Relative integrated density (C3a/GAPDH)
NS
2 days
7 days
12 days
c
IB: Sap2
ISO #1
ISO #2
ISO #3
ISO #4
ISO #5
ISO #6
ISO #7
Sap2-273V
Sap2 1ng
ISO #8
ISO #9
ISO #10
ISO #11
ISO #12
ISO #13
ISO #14
d
Absorbance (450nm)
Sap123 ko
e
C3b deposition
PBS
NHS
f
CFSE-CA: P2
DIO: P2
g
Sap2-273V: P2
Iso #14: P2
FITC-A
APC-A

Figure S3. C3a release in the kidney and the effect of V273L variation of Sap2 on C3b/iC3b deposition and C3b/iC3b mediated phagocytosis of *C. albicans*. (**a-b**) C3a level in the kidney of fungal infected mice. Mice were intravenously infected with Sap2-273L (n=6), Sap2-273V strains (n=6), or PBS (n=3), ($1x10^5$ CFU/mouse). At day 2, day 7, and day 12 post infection, C3a in the kidney was detected by Western blotting and analyzed by integrated density. **(c-d)** Quantification of Sap2 secretion levels in the different enriched culture supernatant of clinical isolates by western blotting (**c**) and ELISA(**d**). (**e)** Analysis of the inhibitory effect on C3b deposition mediated by recombinant $Sap2_{273V}$, and $Sap2_{273L}$, or culture supernatant of Sap2-273L or Sap2-273V strains. (**f**) The representative flow plots of CFSE labeled *C. albican* and DiD labeled PMA-differentiated cells. (**g**) The THP-1 cells were first gated and chosen by FSC-A-FSC-H. Then the percent of DiD and FITC positive cells indicated the percent of phagocytosed cells among all THP-1 cells. Data are shown as means ± SD and from one of three independent experiments. *$P < 0.05$, **$P < 0.01$, Student's *t*-test.

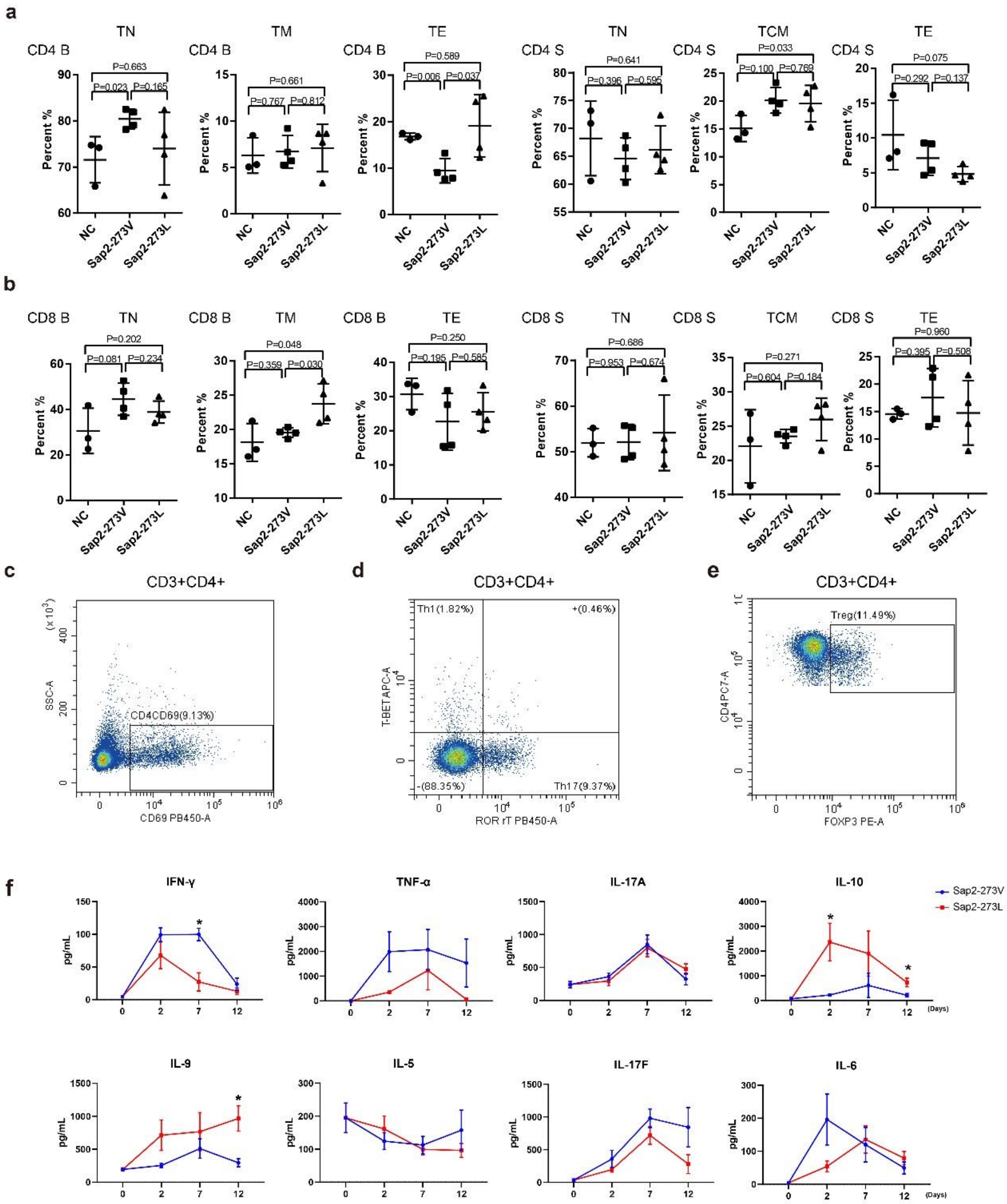
a
CD4 B TN
P=0.663
P=0.023 P=0.165
CD4 B TM
P=0.661
P=0.767 P=0.812
CD4 B TE
P=0.589
P=0.006 P=0.037
CD4 S TN
P=0.641
P=0.396 P=0.595
CD4 S TCM
P=0.033
P=0.100 P=0.769
CD4 S TE
P=0.075
P=0.292 P=0.137
Percent %
NC
Sap2-273V
Sap2-273L
b
CD8 B TN
P=0.202
P=0.081 P=0.234
CD8 B TM
P=0.048
P=0.359 P=0.030
CD8 B TE
P=0.250
P=0.195 P=0.585
CD8 S TN
P=0.686
P=0.953 P=0.674
CD8 S TCM
P=0.271
P=0.604 P=0.184
CD8 S TE
P=0.960
P=0.395 P=0.508
c
CD3+CD4+
SSC-A
CD4CD69(9.13%)
CD69 PB450-A
d
CD3+CD4+
Th1(1.82%)
+(0.46%)
-(88.35%)
Th17(9.37%)
T-BET APC-A
ROR rT PB450-A
e
CD3+CD4+
Treg(11.49%)
CD4PC7-A
FOXP3 PE-A
f
IFN-γ
TNF-α
IL-17A
IL-10
IL-9
IL-5
IL-17F
IL-6
pg/mL
(Days)
Sap2-273V
Sap2-273L

Figure S4. The Sap2-273L triggered different T cell responses. Mice were intravenously infected with the same amount of Sap2-273V (n=4), Sap2-273L strains (n=4), or PBS (n=3), ($1x10^5$ CFU/mouse) for 12 days post infection, (**a** and **b**) Analysis for $CD4^+$/$CD8^+$ T cell differentiation (naïve T, effector T, and memory T cells) according to the expression of CD44 and CD62L in “B” (blood) and “S” (spleen). (**c-e**) The gating strategy Th1, T17, and Treg. All experiments were repeated three times, and one representative experiment is shown. The P values were calculated by T-test. **(f)** The levels of different cytokines released in serum at day 2, day 7, and day 12 post infection. Mice were intravenously infected with the same amount of Sap2-273L (n=6), Sap2-273V strains (n=6) or PBS (n=3), ($1x10^5$ CFU/mouse). The cytokines in serum were detected by Cytometric Bead Array. Data are shown as means ± SD and from one of three independent experiments. *$P < 0.05$, Student’s *t*-test.

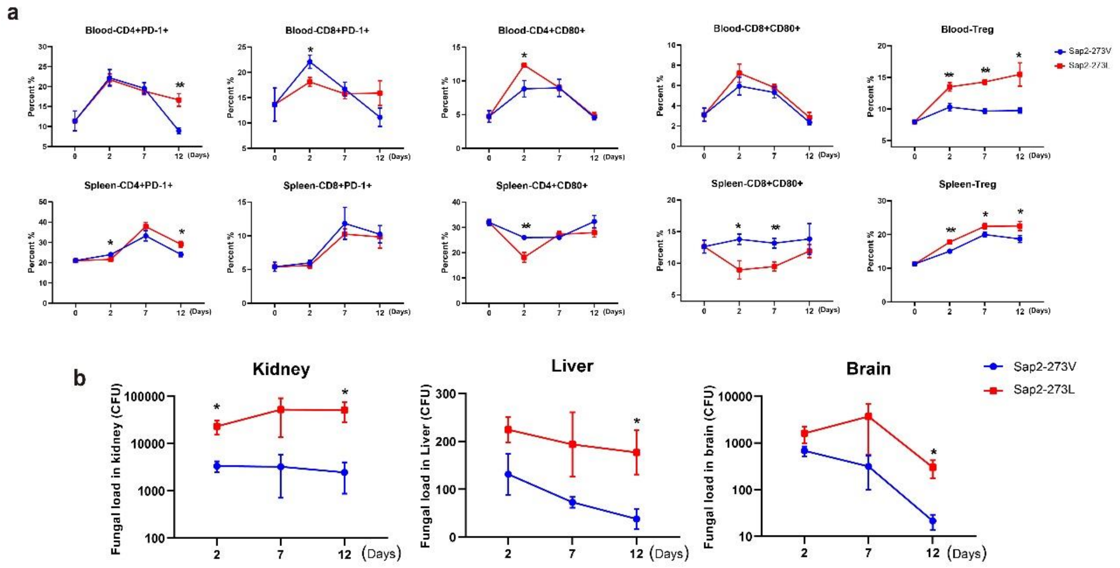

Figure S5. The Sap2-273L triggered different T cell responses and the fungal loads in different organs. (**a**) Dynamic monitoring of activation and exhaustion of CD4^{+}/CD8^{+} T cells in blood and spleen by flow cytometry. The activation and exhaustion of CD4^{+}/CD8^{+}T cells in the blood and spleen were analyzed on day 2, day 7, and day 12 post infection. Mice were intravenously infected with the same amount of Sap2-273L (n=6), Sap2-273V strains (n=6) or PBS (n=3), (1x10^{5} CFU/mouse). (**b**) The fungal loads in different organs on day 2, day 7, and day 12 post infection. Half of the kidney, 0.2 g of the liver, and the whole brain were homogenized and used for plating. Data are shown as means ± SD. *P < 0.05, **P < 0.01, Student's t-test.

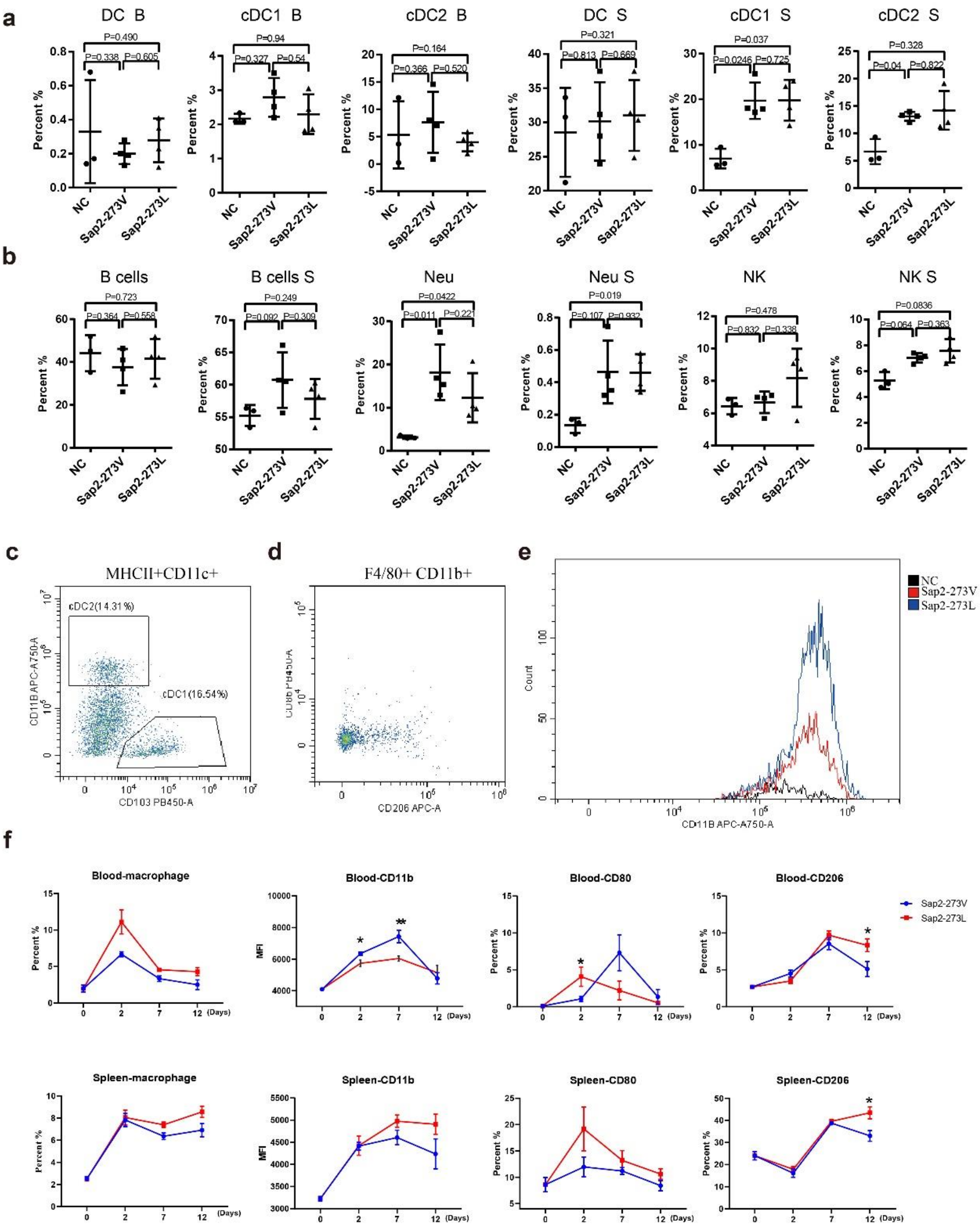
a
DC B
cDC1 B
cDC2 B
DC S
cDC1 S
cDC2 S
b
B cells
B cells S
Neu
Neu S
NK
NK S
Percent %
NC
Sap2-273V
Sap2-273L
c
MHCII+CD11c+
cDC2(14.31%)
cDC1(16.54%)
CD11B APC-A750-A
CD103 PB450-A
d
F4/80+ CD11b+
CD206 APC-A
e
NC
Sap2-273V
Sap2-273L
Count
CD11B APC-A750-A
f
Blood-macrophage
Blood-CD11b
Blood-CD80
Blood-CD206
Spleen-macrophage
Spleen-CD11b
Spleen-CD80
Spleen-CD206
MFI
(Days)

Figure S6. ***C. albicans*** **triggered immunosuppressed T cell responses were caused by induction of macrophage M2-like phenotype. (a-b)** Mice were infected by Sap2-273L (n=4), Sap2-273V strains (n=4), or PBS (n=3), (1x10^5 CFU/mouse). (**a**) Analysis of the cDCs of B (blood) and S (spleen) by flow cytometry according to the expression of CD11b and CD103. **(b)** Analysis of B cells, Neutrophils, and NK of B (blood) and S (spleen). **(c)** The gating strategy for cDC1 and cDC2. **(d)** The gating strategy for M2 macrophage according to the high expression of CD206. **(e)** The types of macrophages in the spleen were examined by flow cytometry using CD11c, CD11b, and F4/80+ as detection markers. **(f)** Dynamic monitoring of macrophage by flow cytometry. Mice were intravenously infected with the same amount of Sap2-273L (n=6), Sap2-273V strains (n=6) or PBS (n=3), (1x10^5 CFU/mouse). Macrophage in blood and spleen was analyzed on day 2, day 7, and day 12 post infection. Data are shown as means ± SD, and from one of three independent experiments. *$P < 0.05$, **$P < 0.01$, Student's *t*-test.

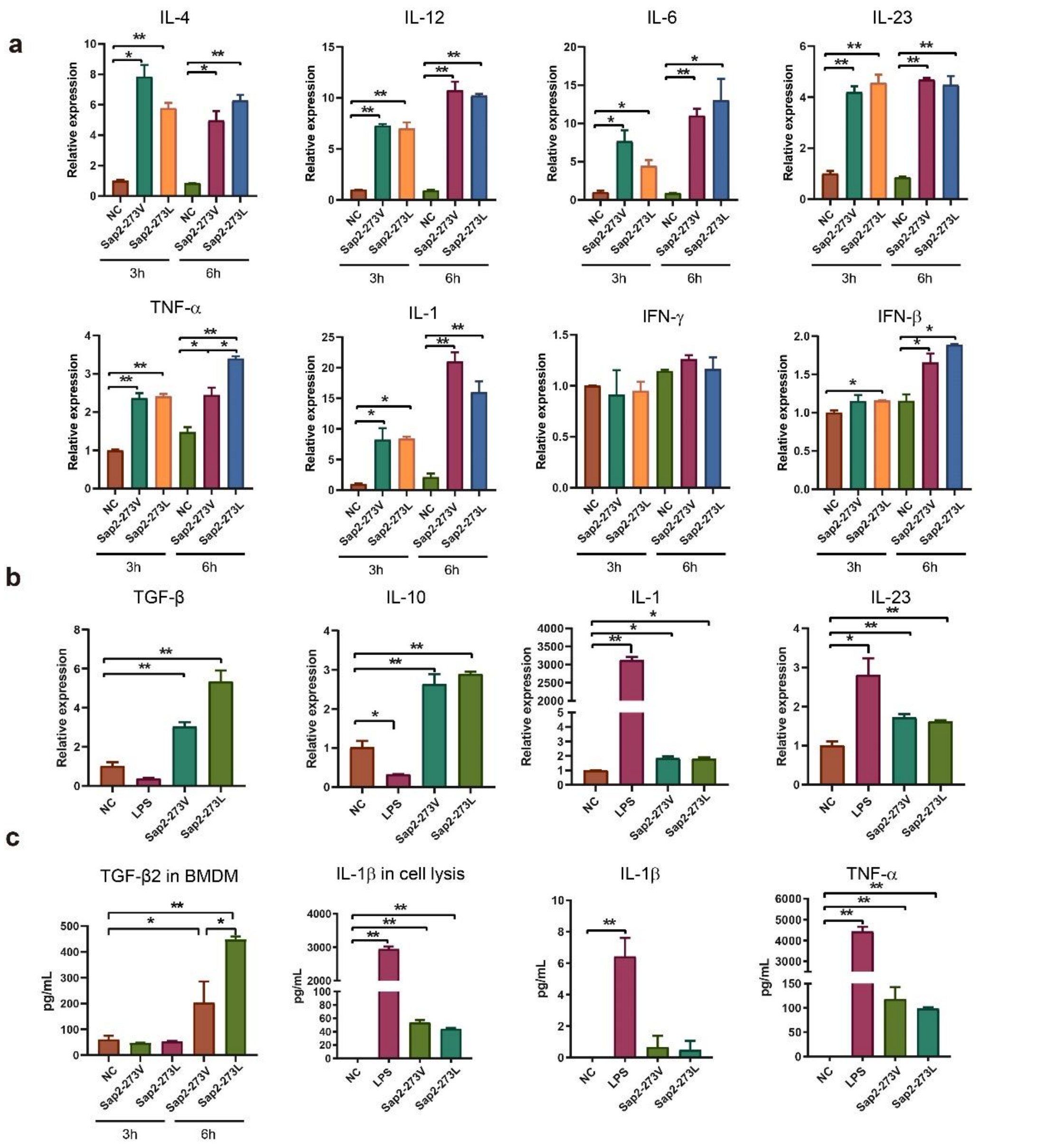

Figure S7. ***C. albicans*** **triggered immunosuppressed T cell responses were caused by induction of macrophage M2-like phenotype. (a)** RAW and BMDM cells were co-cultured with Sap2-273L and Sap2-273V strains for 3h and 6h, cytokines (IL-4, IL-12, IL-6, IL-23, TNF-α, IL-1β, IFN-γ, and IFN-β) were detected by qPCR. **(b)** Cytokine (TGF-β, IL-10, IL-1β, and IL-23) expression was detected in RAW cells by qPCR. **(c)** TGF-β2, IL-1β, and TNF-α in the supernatant and the cell lysate were detected by ELISA. Data are shown as means ± SD. *$P < 0.05$, **$P < 0.01$, Student's *t*-test.

Table S1. Antibodies used in the flow cytometry analysis

	488/561nm				635nm		405nm	
	FITC	PE	Percpcy 5.5	Pe-cy7	APC	APC-CY7	BV421	BV510
B/NK/Neu	CD45		FVS	NK1.1	CD3	Ly-6G	CD19	CD11b
Company-Cat.	BD-553080		BD-564996	BD-552878	BD-553066	BD-560600	BD-562701	BD-562950
T/Treg/TH1/Th17	CD45	FOXP3		CD4	T-bet	CD3e	RoRrT	FVD
Company-Cat.	BD-553080	eB-12-5773-82		Biolegend-100422	Biolegend-644813	Biolegend-100222	BD-562894	Biolegend-423102
Tfh/Tex	CD45		CXCR5	CD4	PD-1	CD3e	CD8a	FVD
Company-Cat.	BD-553080		BD-560528	Biolegend-100422	Biolegend-135210	Biolegend-100222	Biolegend-100725	Biolegend-423102
M/1/2	CD45	F4/80		CD11c	CD206	CD11b	CD86	FVD
Company-Cat.	BD-553080	BD-565410		BD-558079	Biolegend-141708	BD-557657	Biolegend-105022	Biolegend-423102
DCs/cDC1/cDC2	CD45		MHCII		CD11c	CD11b	CD103	FVD
Company-Cat.	BD-553080				Biolegend-117310	BD-557657	BD-566297	Biolegend-423102
T cells activation	CD45	CD62L	CD8		CD44	CD4	CD69	FVD
Company-Cat.	BD-553080	BD-553151	BD-551162		eB-17-0441-82	BD-552051	Biolegend-104527	Biolegend-423102

Table S2. Primers for different cytokines analysis by qPCR

Target gene	F(5'-3')	R(5'-3')
Actin	CATTGCTGACAGGATGCAGAAGG	TGCTGGAAGGTGGACAGTGAGG
Tgfb1	TGATACGCCTGAGTGGCTGTCT	CACAAGAGCAGTGAGCGCTGAA
Tgfb2	TTGTTGCCCTCCTACAGACTGG	GTAAAGAGGGCGAAGGCAGCAA
Tgfb3	AAGCAGCGCTACATAGGTGGCA	GGCTGAAAGGTGTGACATGGAC
Tnfa	GGTGCCTATGTCTCAGCCTCTT	GCCATAGAACTGATGAGAGGGAG
Ifnγ	CAGCAACAGCAAGGCGAAAAAGG	TTTCCGCTTCCTGAGGCTGGAT
Ifnb	GCCTTTGCCATCCAAGAGATGC	ACACTGTCTGCTGGTGGAGTTC
Il-1b	TGGACCTTCCAGGATGAGGACA	GTTCATCTCGGAGCCTGTAGTG
Il-4	ATCATCGGCATTTTGAACGAGGTC	ACCTTGGAAGCCCTACAGACGA
Il-6	TACCACTTCACAAGTCGGAGGC	CTGCAAGTGCATCATCGTTGTTC
Il-10	CGGGAAGACAATAACTGCACCC	CGGTTAGCAGTATGTTGTCCAGC
Il-12	TTGAACTGGCGTTGGAAGCACG	CCACCTGTGAGTTCTTCAAAGGC
Il-23	CATGCTAGCCTGGAACGCACAT	ACTGGCTGTTGTCCTTGAGTCC
